# Permanent cilia loss during cerebellar granule cell neurogenesis involves withdrawal of cilia maintenance and centriole capping

**DOI:** 10.1073/pnas.2408083121

**Published:** 2024-12-20

**Authors:** Sandii Constable, Carolyn M. Ott, Andrew L. Lemire, Kevin White, Yu Xun, Amin Lim, Jennifer Lippincott-Schwartz, Saikat Mukhopadhyay

**Affiliations:** ^a^Department of Cell Biology, University of Texas Southwestern Medical Center, Dallas, TX 75390; ^b^Janelia Research Campus, Howard Hughes Medical Institute, Ashburn, VA 20147

**Keywords:** cilia disassembly, intraflagellar transport, granule cell, medulloblastoma, Sonic hedgehog

## Abstract

Brain neurons utilize the primary cilium as a privileged compartment to detect and respond to extracellular ligands such as Sonic hedgehog (SHH). However, cilia in cerebellar granule cell (GC) neurons disassemble during differentiation through ultrastructurally unique intermediates, a process we refer to as cilia deconstruction. In addition, mature neurons do not reciliate despite having docked centrioles. Here, we identify molecular changes that accompany cilia deconstruction and centriole docking in GC neurons. We used single cell transcriptomic and immunocytological analyses to compare the transcript levels and subcellular localization of proteins between progenitor, differentiating, and mature GCs. Differentiating GCs lacked transcripts for key activators of premitotic cilia resorption, indicating that cilia disassembly in differentiating cells is distinct from premitotic cilia resorption. Instead, during differentiation, transcripts of many genes required for cilia maintenance—specifically those encoding components of intraflagellar transport, pericentrosomal material, and centriolar satellites—decreased. The abundance of several corresponding proteins in and around cilia and centrosomes also decreased. These changes coincided with downregulation of SHH signaling prior to differentiation, even in a mutant with excessive SHH activation. Finally, mother centrioles in maturing granule neurons recruited the cap complex protein, CEP97. These data suggest that a global, developmentally programmed decrease in cilium maintenance in differentiating GCs mediates cilia deconstruction, while capping of docked mother centrioles prevents cilia regrowth and dysregulated SHH signaling. Our study provides mechanistic insights expanding our understanding of permanent cilia loss in multiple tissue-specific contexts.

## Significance

Sonic hedgehog (SHH) promotes proliferation of granule cell (GC) progenitors in the postnatal cerebellum and in the pediatric tumor, medulloblastoma, through specialized compartments called primary cilia. During neuronal maturation, GCs stop proliferating and deconstruct cilia. Here, we found that depletion of transcripts and decreased ciliary and periciliary localization of proteins required for cilium and centrosome maintenance coincided with cilium deconstruction and downregulation of SHH signaling. Furthermore, we noted capping of mother centrioles following cilia loss, which likely prevented reciliation. Our results suggest that development of interventions promoting cilia deconstruction and centriole capping could restrict SHH responsiveness in treatment of medulloblastoma. In other tissues, cilia deconstruction might also regulate cellular responsiveness to developmental cues.

Primary cilia are microtubule-based structures templated from mother centrioles that function as signaling hubs in diverse cellular contexts (1–4). Although many neurons are ciliated (5–8), cerebellar granule cell (GC) neurons require cilia prior to differentiation, but disassemble them as they mature (9–12). Recent analysis of volumetric electron microscopy (EM) of postnatal mouse cerebellum revealed a unique disassembly mechanism—called cilia deconstruction—which included intermediate structures similar to those observed during ciliogenesis (12). The ultrastructural analysis also revealed that mother centrioles moved to the plasma membrane where distal appendages docked, but cilia did not regrow (12). Aberrant growth of GCs causes medulloblastoma, the most common malignant pediatric brain tumor (13). Given that subtypes of medulloblastoma cells resemble GC progenitors and regain both cilia and responsiveness to the mitogen Sonic hedgehog (SHH) (14–17), understanding cilia deconstruction could reveal molecular targets to disrupt tumor growth.

**Fig. 1.**
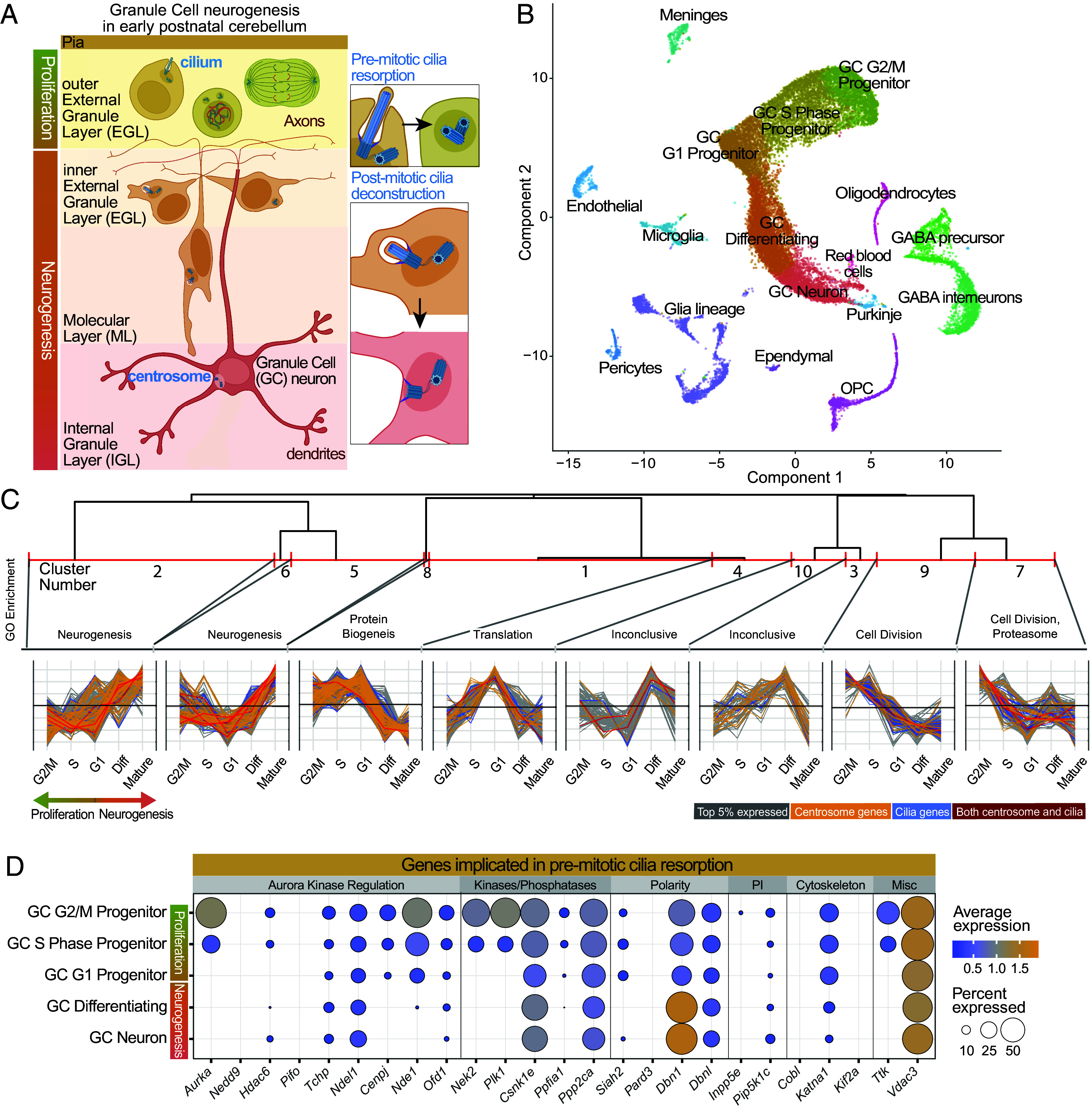
Transcriptomic differences between proliferating and differentiating GCs suggest unique regulation of cilia deconstruction in postmitotic cells. (*A*) During neurogenesis, progenitor GCs proliferate in the outer EGL. Differentiating GCs begin in the inner EGL and migrate to the IGL as they mature. Cilia present on GCs in the outer EGL resorb cilia prior to mitosis. Cilia of differentiating GCs are deconstructed during neuronal maturation (12). The mother centriole of mature GCs dock at the plasma membrane without extending a cilium (12). (*B*) scRNA-seq datasets from P5, P7, and P14 mice (26) were combined, and cells were clustered based on differential expression. Clusters are plotted as a Uniform Manifold Approximation and Projections (UMAP) and the cell types are labeled. (*C*) The expression of the top 5% of detected transcripts combined with a curated list of cilia and centrosome genes was normalized and genes were clustered based on expression pattern across the GC lineage. The dendrogram of clusters is shown at the top. The red line represents cutoff value that created the 10 clusters. The clusters were analyzed using DAVID (27) and similar, highly significant GO terms were used to label the clusters. The normalized expression pattern of each gene in each cluster is graphed. Genes from the top 5% are gray, centrosome genes are orange, cilia genes are blue and genes that are both cilia and centrosome proteins are red. (*D*) Expression of genes known to be involved in premitotic cilia disassembly or severing were plotted for each cluster in the GC lineage. Transcript detection frequency is indicated by the dot size and dot color represents the average expression level.

The regulation of cilia disassembly has previously been studied in cultured cells prior to cell division (18–20). However, we recently reported that cilia deconstruction during GC neurogenesis occurs in postmitotic differentiating cells (12). It is unclear whether the same molecular pathways govern ciliary disassembly in dividing and postmitotic cells. Also, in contrast to cultured cells, GC cilia deconstruction occurs in a complex multicellular environment in the developing cerebellum (12). GC progenitors proliferate in response to coincidental inputs from SHH, and laminin present in the extracellular matrix close to the pia (9). Upon differentiation, dramatic reprogramming propels GCs along glial projections away from the pia (21). Completion of GC neuronal maturation upon arrival at the internal granule layer (IGL) involves dendritic maturation and establishment of synaptic connections (22). Progenitor cells in the outer external granule layer (EGL) all have cilia (14, 15), which we recently determined are resorbed prior to mitosis and then reassembled after mitosis (12). We also found that in progenitor cells that had stopped dividing and begun to differentiate, cilia were deconstructed either before or as GCs migrated from the inner EGL through the molecular layer (ML) to the IGL (Fig. 1*A*) (12). The molecular mechanisms causing primary cilia deconstruction in differentiating GCs remain unclear, including whether any known premitotic cilia resorption regulators are involved (23–25). In addition, mechanisms that prevent cilia reassembly from docked centrioles in mature GC neurons have not been identified.

To investigate the molecular mechanisms that accomplish cilia deconstruction and prevent reciliation in differentiating GCs, we used single cell transcript analysis and immunocytochemistry. Several components required for premitotic cilia resorption were transcriptionally undetectable in differentiating GCs indicating that distinct mechanisms regulated cilia disassembly in postmitotic neurons. Concurrently, multiple cilia and centrosome transcripts decreased and localization of the corresponding proteins in and around cilia was lost. In addition, we found that a centriole capping protein was progressively recruited to mother centrioles as GC neurons matured. Regulated cilia deconstruction coincided with the loss of SHH responsiveness during GC differentiation. It is known that loss of individual cilia or centrosome proteins can cause pathological cilia disassembly (23–25). Our study suggests that cilia disassemble during GC neurogenesis because factors required for cilia maintenance are withdrawn through coordinated global reductions in both transcript levels and protein localization to cilia and centrosomes. In mature GCs, cilia regrowth could be prevented both by the absence of necessary factors and by capping of docked mother centrioles.

## Results

### Global Transcriptional Changes Include Downregulation of Cilia and Centrosome Genes During GC Neurogenesis.

Dramatic transcriptional reprogramming occurs when GC progenitors begin differentiating (28, 29). To identify molecular regulators of cilia deconstruction we compared transcript levels in progenitor and differentiating GCs utilizing previously published single cell transcriptomic data of developing mouse cerebella (26). We pooled the P5, P7, and P14 datasets, clustered cells by similarity and assigned identities based on expression of established marker genes (21) (Fig. 1*B*, *SI Appendix*, Fig. S1, and Dataset S1). In the UMAP plot (Fig. 1*B*), the GC progenitor and mature cells were a continuum separated into five clusters based on gene expression: G1, S phase, and G2/mitosis progenitors, differentiating neurons and mature neurons. G1 progenitors, which can divide or differentiate, were in the center with the cell cycle progression clusters and the differentiation and maturation clusters extending in different directions. The expression of markers for each GC cluster is presented in *SI Appendix*, Fig. S2*A*.

To identify potential regulators of cilia deconstruction we normalized gene expression levels within the five GC clusters and created expression trendlines. We then performed cluster analysis using the top 5% of genes (1,399 genes) detected in the GC lineage to group genes with similar expression patterns. The cutoff value (k value) of 9 in the resulting dendrogram provided broad separation of gene expression groups (*SI Appendix*, Fig. S2*B* and Dataset S2). Gene ontology (GO) analysis using DAVID (27) revealed pathway component enrichment in each expression cluster (Dataset S3 and *SI Appendix*, Fig. S2*C*; labels are based on highly represented GO terms). Although this strategy captured major trends (e.g. decreases in cell cycle components, increases in neuron-specific proteins) no centrosome or cilia-related GO terms were enriched in any of the clusters, although individual genes with cilia or centrosome GO terms were present (*SI Appendix*, Table S1). Because cilia and centrosomes are low-frequency organelles, and scRNASeq has limited read depth, we were concerned that the short list of highly expressed genes might not suffice for the analysis. However, assessing a larger percentage of genes resulted in more crowded cluster dendrograms with less useful clusters.

To work around these issues, we created a curated list of genes that included cilia and centrosome proteins and regulatory factors (Dataset S4 and *SI Appendix*, Table S2). Similar gene expression pattern clustering performed with the added curated genes and a k value of 10 provided similar cluster separation to k value of 9 for the top 5% of GC transcripts. Fig. 1*C* shows the top of the dendrogram and the normalized expression patterns (genes in each cluster are listed in Dataset S5). DAVID analysis revealed cilia and centrosome GO terms enriched in each cluster (Dataset S6). Several GO terms were enriched both in clusters with increasing expression and in clusters with decreasing expression (e.g. centrosome, cilium, cilium assembly, ciliary basal body). However, GO terms for intraciliary transport, intraciliary retrograde transport, intraciliary transport particle B, and ciliary transition zone, as well as centriolar satellites and pericentriolar material (PCM) were only in clusters with lower expression in mature neurons (Clusters 1 and 9). These were noteworthy because centriolar satellites and PCM proteins regulate ciliogenesis and promote ciliary maintenance (30). Intraciliary transport, also called intraflagellar transport (IFT), which moves cargo to and within cilia is required for cilia maintenance (30). Transcripts of the centriolar satellite protein, *Pcm,1* and the IFT protein, *Ift27,* which were both included in the top 5% of transcripts (*SI Appendix*, Table S1), clustered with genes whose expression decreased during differentiation and maturation in both analyses (Datasets S3 and S5). Thus, instead of identifying candidate regulatory factors, the expression pattern clustering suggested that widespread transcriptional changes included downregulation of genes that contribute to cilia maintenance.

### Key Premitotic Cilia Disassembly Regulators Were Transcriptionally Downregulated During Postmitotic Cilia Deconstruction.

We next examined expression of individual transcripts by generating dot plots of gene expression for both the curated list and the top 5% (Dataset S7). Known premitotic cilia disassembly-promoting genes were detected in the S and G2/M clusters, some at high frequency (Fig. 1*D*) as expected because these cells disassemble cilia prior to mitosis (12). In differentiating and mature GCs, which undergo postmitotic cilia deconstruction, expression of many of these transcripts decreased. For example, Aurora kinase A, essential for promoting cilia disassembly (18, 20), was detected only in S and G2/M progenitors. Factors that interact with or are regulated by AURKA, *Hef1* (*Nedd9*), and *Pifo* (*Pitchfork*) (23–25), were not detected above threshold and *Tchp* (*trichoplein*) expression fluctuated slightly. *Hdac6* diminished during differentiation and then increased in mature cells [possibly related to the pervasive acetylated microtubules throughout neurons (31)]. Expression of *Cenpj, Nde1, and Ofd1*, components of a cilium disassembly complex (32), also decreased in differentiating and mature GCs. Additional kinases undetected in differentiating and mature GCs included the NIMA-related kinase, *Nek2* (33), the polo-like kinase 1 (*Plk1*) (34), and *Ttk* (35) (Fig. 1*E*). Expression of the microtubule depolymerizing kinesin *Kif2a*, a target of PLK1 (36), also decreased in differentiating and mature GCs but to a lesser extent. Expression of *Siah2* and *Pard3*, polarity genes that influence cilia maintenance and cilia ectosome shedding (9, 37), were highest in proliferating GC progenitors. In contrast, *Debrin* (*Dbn1*) and *Debrin-like* (*Dbnl*) expression did not decrease, possibly because they participate in nucleokinesis and migration (38).

Another mechanism for cilia removal in response to stress, pharmacological induction, or serum addition is rapid shedding of cilia (25, 39). Expression of *Katna1,* the gene encoding the microtubule severing protein katanin (39), diminished in differentiating and mature GCs (Fig. 1*D*). This lack of evidence for severing is consistent with the observation of only a single cilium with constriction suggestive of severing (12). We also examined the levels of *Inpp5e*, a phosphoinositide 5’phosphatase involved in the decapitation of ciliary tips (37), which was only detected in G2/M GCs (Fig. 1*D*). In summary, transcripts for proteins that promote cilia resorption were largely reduced or undetected in differentiating and mature GCs, although they were present in cycling progenitor cells. Thus the reported mechanisms of premitotic cilia disassembly were not likely driving cilia deconstruction in postmitotic GC neurons.

### IFT Transcripts and Proteins Decreased Upon GC Maturation.

**Fig. 2.**
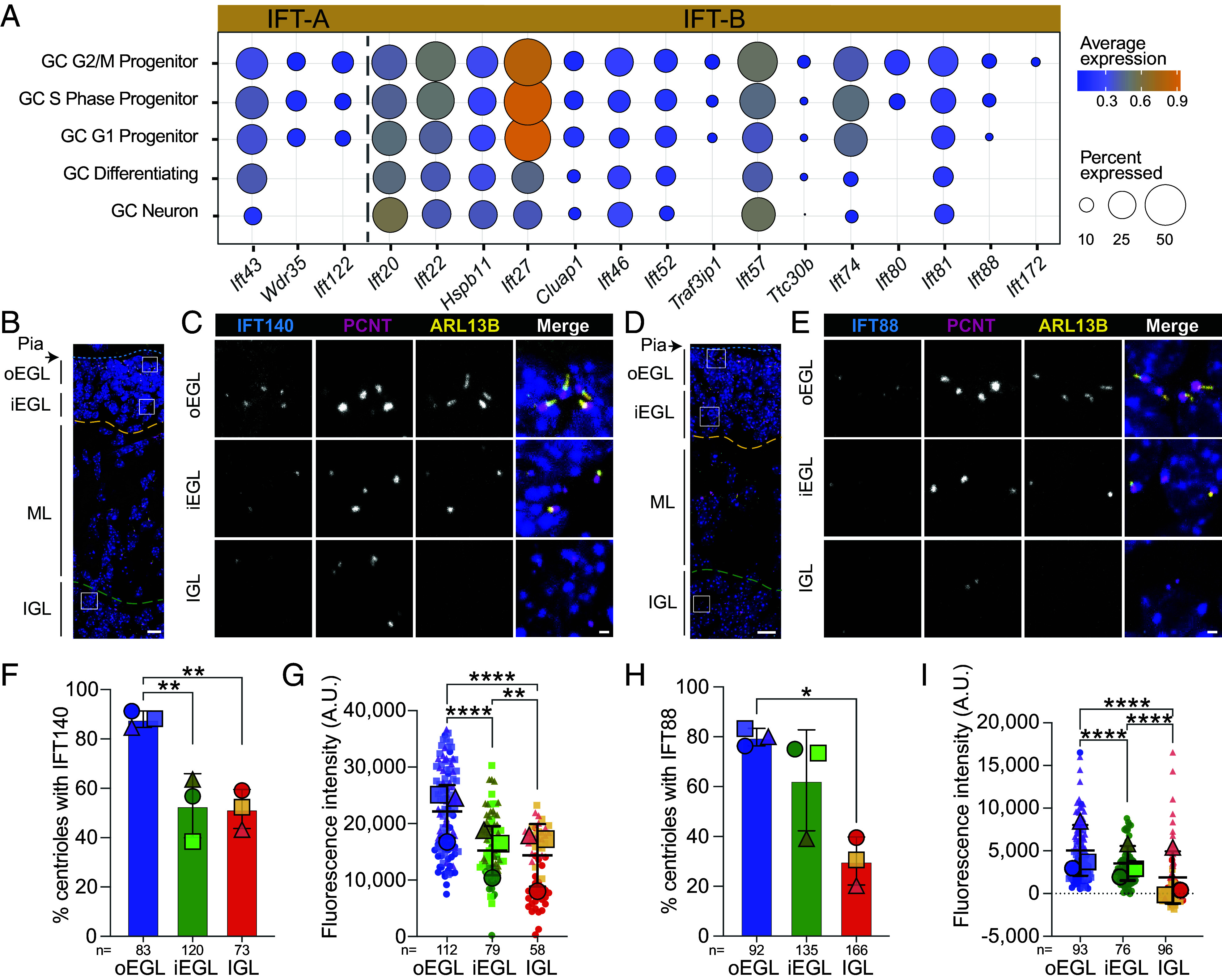
IFT transcripts and proteins decreased upon GC maturation. (*A*) Expression level and detection frequency of IFT genes detected above the 5% threshold are plotted for each GC cell cluster. Transcript detection frequency is indicated by the dot size and dot color represents the average expression level. (*B*–*E*) Sagittal section of the P7 mouse cerebellum stained with antibodies to the indicated proteins and costained with DAPI (dark blue) were imaged by confocal fluorescence microscopy. Lines mark Pia (blue dashed line), EGL (between blue line and yellow dashed line), ML, and IGL (green dashed line). The indicated regions of the outer EGL, inner EGL, and IGL in *B* and *D* are magnified in *C* and *E*, respectively. (*F*–*I*) Sections from three individual animals were stained and imaged. The percent of PCNT positive centrioles that also had IFT40 (*F*) or IFT88 (*H*) signal is graphed. In addition, the fluorescence intensity of each IFT40 (*G*) or IFT88 (*I*) puncta was quantified after background subtraction and the values are plotted for each layer of the developing cerebellum. Statistical analysis was performed using multiple comparison ANOVA. A.U.= arbitrary units; scale bar: 10 μm (*B* and *D*), 1 μm (*C* and *D*).

The gene expression clustering indicated that expression of IFT, PCM, and centriolar satellite components was downregulated as GCs differentiated. To investigate this further, we assessed expression of the IFT-A and IFT-B complex proteins, which regulate retrograde and anterograde transport in cilia (40). The IFT-A complex also regulates preciliary cargo trafficking (41). Loss of key proteins from either complex causes cilia disassembly (30). We found only three genes coding for IFT-A components detected above the threshold (5% of cells in the cluster). Of these, *Ift43* expression was decreased in mature neurons and *Wdr35* and *Ift22* were undetectable after the onset of differentiation (Fig. 2*A*). IFT-B component genes showed decreased expression in differentiating and/or mature GC neurons for 10 of the 15 genes, indicating global downregulation of IFT component proteins during neural maturation. It was notable that *Ift20, 2410089E03Rik (Cplane1*/*Jbts17), Cep19,* and *Rabl2* (42) expression did not diminish like other IFT components (Fig. 2*A*). IFT20 promotes trafficking to docked centrioles at the immune synapse (43). While it is possible these proteins have other roles, we anticipate that they function at docked centrioles in adult GCs.

Transcriptional programs participate in GC differentiation (28, 29); however, protein levels and localization more directly represent cellular activities. To investigate IFT protein expression and localization, we stained P7 cerebella sections with antibodies to the IFT-A protein, IFT140, or the IFT-B protein, IFT88 (Fig. 2 *B*–*E*). GC populations at different developmental stages were distinguished based on cell location (Figs. 1 *A* and 2 *B*) (21). Sections were also stained with antibodies to the cilia marker, ARL13B, and the centrosome marker, pericentrin (PCNT). IFT88 and IFT140 both localized adjacent to centrosomes (Fig. 2 *C* and *D*). We quantified both the frequency of IFT protein localization adjacent to the centrosome and the fluorescence intensity. The percentage of PCNT positive centrosomes costained with IFT140 decreased after differentiation began (Fig. 2*F*). IFT88 puncta in the outer and inner EGL were similar, however, in the IGL where cilia were sparse, IFT88 detection decreased (Fig. 2*H*). The total fluorescence intensity of IFT140 decreased significantly between the outer EGL and the inner EGL and remained low in the IGL (Fig. 2*G*). IFT88 intensity was similar in the outer and inner EGL but was barely detectable above background levels in the IGL (Fig. 2*I*). Although more intense than IFT proteins, the frequency and intensity of PCNT also diminished in the IGL as discussed in the next section. Together, the transcriptional changes in IFT correlated with reduced centrosome recruitment of the tested IFT proteins as GCs differentiated and migrated.

### PCM Transcripts and Proteins Are Reduced During GC Differentiation.

**Fig. 3.**
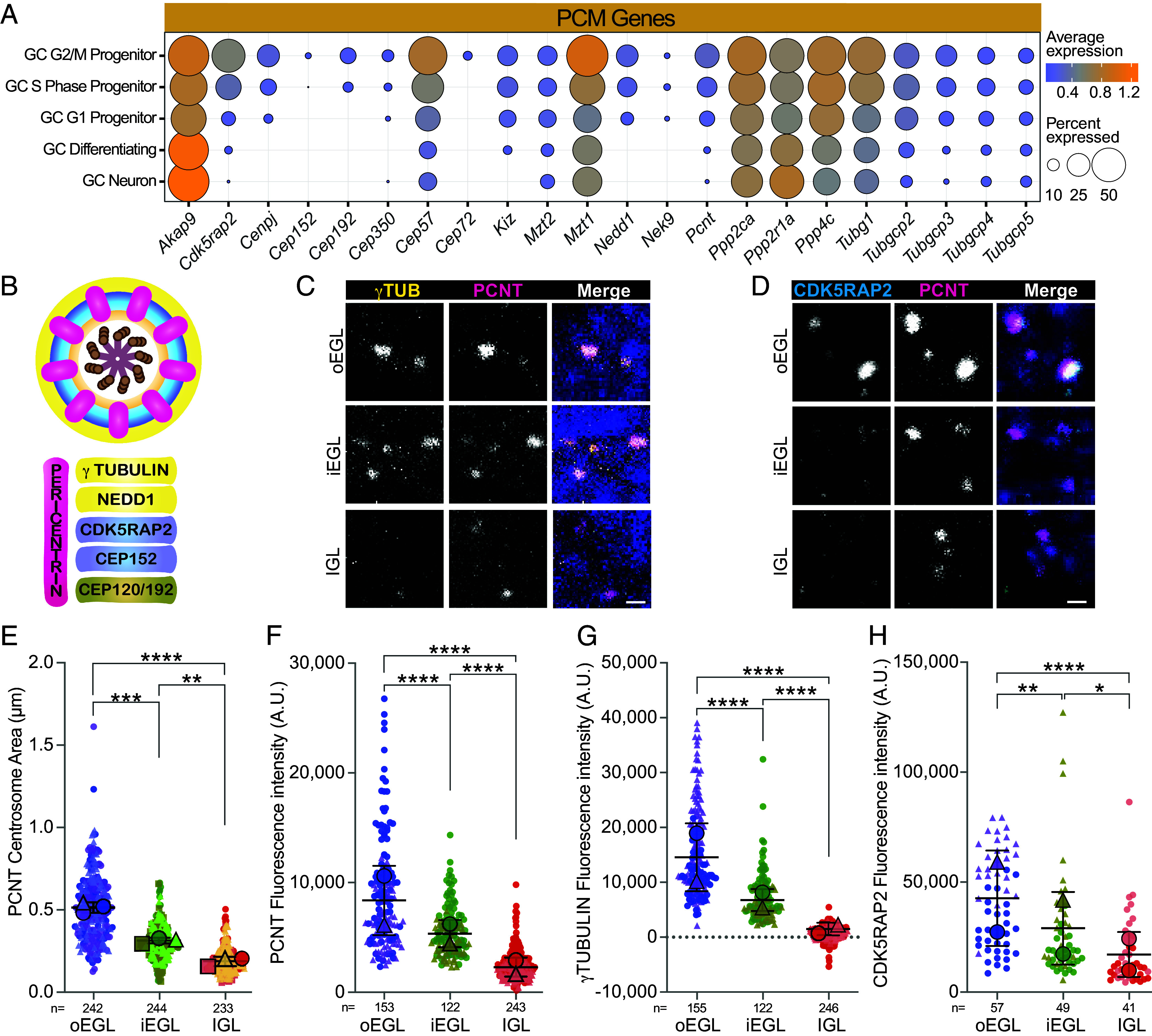
PCM transcripts and proteins are reduced during GC differentiation. (*A*) The average expression level and detection frequency of the indicated PCM genes are plotted for each GC cluster. Transcript detection frequency is indicated by the dot size and dot color represents the average expression level. (*B*) A representative drawing of a centrosome cross section illustrating layers of PCM. Schematic adapted from (44). (*C* and *D*) Sagittal sections of P7 mice cerebellum were stained with the indicated antibodies to PCM proteins and costained with DAPI (dark blue) followed by confocal microscopy. The representative images are from the outer EGL, inner EGL, and IGL as indicated. (*E*) The area of each PCNT puncta was measured and is plotted by cerebellum layer from three sections from each of 2 to 3 individual animals. The average measurements from each animal were superimposed as large symbols. (*F*–*H*) The fluorescent intensity of PCNT (*F*), γtubulin (*G*), and CDK5RAP2 (*H*) puncta in the indicated layers are plotted. Statistical analysis was preformed using multiple comparison ANOVA. A.U.= arbitrary units; scale bar: 1 μm.

To investigate possible reductions in PCM during GC neuronal maturation, we examined both expression and localization patterns. Transcription of 17 of the 22 PCM genes detected above threshold decreased during differentiation and/or neuronal maturation (Fig. 3*A*). The gene coding for PCNT, *Pcnt,* a PCM spoke protein that determines the PCM circumference (Fig. 3*B*) (44), was among the genes with decreased expression as neurogenesis progressed. PCNT was detected in all layers, however, protein levels diminished during neuronal maturation (Fig. 3 *C* and *D*). Both the total area and the fluorescence intensity progressively decreased (Fig. 3 *E* and *F*). Antibody accessibility was similar throughout the tissue sections as evidenced by the high PCNT fluorescence intensity in SOX9+ glia (45) (*SI Appendix*, Fig. S3). Thus, both PCNT transcript and protein levels diminished as GC neuronal maturation progressed.

*Tubg1*, which codes for γ–tubulin, several genes coding for γ–tubulin ring complex (γ–TURC) components (46), and *Nedd1 and Cdk5rap2*, are all detected in fewer cells and at lower levels and/or less frequently in the differentiating and mature GCs (Fig. 3*A*). These proteins, which contribute to microtubule nucleation (47, 48) and the centrosomal microtubule organizing center, form the outer layer of the PCM (Fig. 3*B*) (44). In the EGL, protein levels of both γ–tubulin and CDK5RAP2 colocalized with PCNT (Fig. 3 *C* and *D*). However, the fluorescence intensity of γ–tubulin and CDK5RAP2 in the inner EGL decreased and became almost nondetectable in the IGL (Fig. 3 *G* and *H*). Thus, like the transcripts, γ–tubulin and associated proteins were largely absent from the mature GCs in the IGL. Among genes detected in GC neurons were the PP2A phosphatase catalytic subunit *Ppp2ca* and regulatory subunit *Ppp2r1a.* The PP2A phosphatase has been implicated in PCM dissolution (49).

While the PCM cannot be visualized by EM, centrioles and distal appendages remained grossly intact during cilia deconstruction (12). Transcription of the genes coding for both the distal centriolar protein, TALPID3 (*2700049A03Rik)*, (50) and the distal appendage protein, CEP164 (*Cep164*) (51), remained in all GCs (Dataset S7). We imaged P7 cerebella sections stained with antibodies to TALPID3 or CEP164 along with anti-PCNT and anti-ARL13B. Both TALPID3 and CEP164 were detected at every developmental stage (*SI Appendix*, Fig. S4). Thus, the size and composition of the PCM diminished during differentiation and maturation, while the centrioles remained intact.

### Centriolar Satellite Protein Expression was Restricted to the Outer Layer of EGL.

**Fig. 4.**
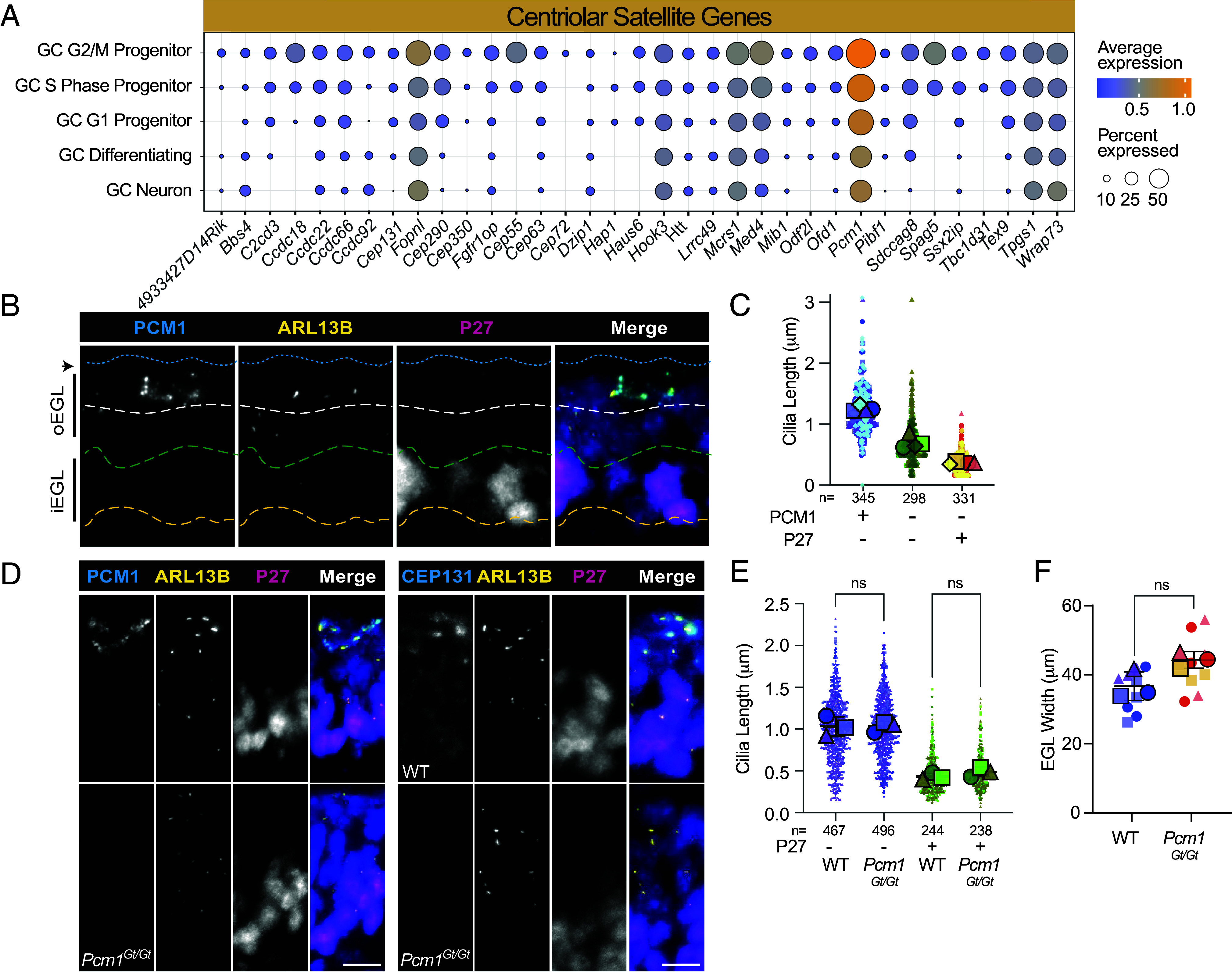
Reduction in centriolar satellite transcripts and proteins in GCs coincided with decreases in ciliary length in the inner EGL. (*A*) The average expression level and frequency of transcript detection for the indicated centriolar satellite genes in each GC cluster was plotted. Transcript detection frequency is indicated by the dot size and dot color represents the average expression level. (*B*) Sagittal sections of P7 cerebella were stained with the indicated antibodies. P27^KIP1^ is a marker of GC differentiation. The blue dashed line indicates the pial surface, the white line is the lower boundary of PCM1 positive cells, the green line is the upper boundary of the P27^KIP1^ positive cells, and the yellow dashed line indicates the inside edge of EGL. (*C*) Cilia lengths were measured in three sections from each of three animals. Lengths were plotted based on the expression of PCM1 and P27^KIP1^ and the average cilium length for each animal is indicated as a large shape. (*D*) Sagittal sections of P8 cerebella from WT or *Pcm1^Gt/Gt^* littermates stained with antibodies to PCM1, ARL13B, and P27^KIP1^, and counterstained with DAPI before imaging using widefield microscopy. (*E*) Cilia lengths were measured in three sections from each of three animals per group at P8. Lengths were plotted based on the expression of P27^KIP1^ and the average cilium length for each animal is indicated as a large shape. Analyzed using multiple comparison ANOVA. (*F*) Width of EGL was measured in five different places in lobes 8 to 10 on three images each from three animals. Each point represents the average of five measurements for each section. Means from each animal are shown as a larger shape. In all merged image panels nuclei stained with DAPI are blue. Scale bar: 10 μm, zoom 1 μm.

The GO term centriolar satellite was identified in clusters with decreased expression in differentiating and mature GCs (Clusters, 1, 7, and 9; Fig. 1*C* and Dataset S6). Centriolar satellites maintain centrosome homeostasis, regulate ciliogenesis, and traffic proteins to the cilium (52). Ciliation decreases upon downregulation of centriolar satellite proteins in cultured cells (53). Thus, we examined expression of individual components: 25 of the 35 centriolar satellite genes (~70%) were detected at lower levels or with lower frequency in differentiating and/or mature GC neurons (Fig. 4*A*). Genes affected included *Pcm1,* the founding centriolar satellite scaffolding protein required for centriolar satellite structure and function (50) and *Cep131*, a gene known to be transcriptionally regulated during cerebellum development by the transcription factor ATOH1 (10). Other notable genes with decreased transcription included *Ofd1*, *Cep290*, *Mib1*, *Sdccag8*, and *Odf2l*. To determine whether downregulated transcription coincided with diminished protein levels, we stained cerebella sections with antibodies to PCM1 or CEP131 along with anti-PCNT and anti-ARL13B antibodies. Centriolar satellites positive for PCM1 or CEP131 were found only in the outer-most layer of the EGL (*SI Appendix*, Fig. S5). We next stained cerebella sections with antibodies to P27^KIP1^, the marker of GC differentiation (Fig. 4*B*). We found two layers of P27^KIP1^ negative cells in the outer EGL: an outer layer that expressed PCM1 and an intermediate layer without PCM1 signal. We compared cilia lengths and found shorter cilia in the PCM1/P27^KIP1^ negative progenitors than in the progenitors with centriolar satellites (Fig. 4 *B* and *C*). Cilia in P27^KIP1^ positive cells of the inner EGL were even shorter. Thus, loss of centriolar satellites correlated with early decreases in cilia length.

To investigate whether loss of PCM1 prevented all cilia formation, we utilized homozygous mice with a gene trap insertion allele of *Pcm1* (*Pcm1^Gt/Gt^*) (54). These mice lack detectable PCM1 protein (54) and we confirmed loss of PCM1 by immunostaining (Fig. 4*D*). CEP131 staining demonstrated lack of centriolar satellites in the *Pcm1^Gt/Gt^* cerebellar GCs (Fig. 4*D*). Loss of PCM1 causes age-dependent cilia abnormalities in a subset of brain regions (54). In developing cerebellar tissue, however, GC progenitors still formed ARL13B positive cilia in the absence of PCM1 (Fig. 4*D*). Cilia length was similar in *Pcm1^Gt/Gt^* pups and wild-type (WT) littermates at each stage of GC differentiation (Fig. 4*E*). The loss of centriolar satellites did not grossly appear to affect cerebellar development (Fig. 4*F*), however, two of the three *Pcm1^Gt/Gt^* pups were slightly smaller than their WT littermates. While not equivalent to the acute loss of PCM1 in differentiating GCs, the absence of centriolar satellites prevented neither cilia formation in progenitor cells nor cilia deconstruction in differentiating GCs. These data suggest that cilia deconstruction is not the result of loss of a single component, but rather a consequence of simultaneous diminishment of several essential components.

### SHH Signaling Downregulation During GC Differentiation Coincided with Ciliary Loss.

Synchronized downregulation of diverse cilia components could be orchestrated by the dramatic transcriptional reprograming that occurs at the onset of differentiation. In progenitor cells, SHH promotes proliferation by activating transcription factors including cyclin D1 and N-MYC, which directly drive the cell cycle (55). Upon onset of differentiation, loss of SHH pathway activation was evident in the scRNA-seq data: expression of *Ccnd1, Gli1, Gli2, Hhip1, Mycn, Ptch1, Ptch2,* and *Sfrp1* (genes directly regulated by SHH pathway transcription factors) decreased (*SI Appendix*, Fig. S6*A*, *Top Left*) (56). Transcription of additional SHH regulating factors (such as *Boc*, *Smo*) also changed, while genes coding for negative regulators of the SHH pathway, such as *Tulp3* (57), *Ankmy2* (58), *Sufu* (59), and PKA regulatory subunits *Prkaca/Prkacb* (60), persisted during neurogenesis (*SI Appendix*, Fig. S6*A*, *Top Right*). Expression of several SHH pathway–associated genes also decreased (*SI Appendix*, Fig. S6*A*, *Bottom*). Expression of *Atoh1,* a transcription factor responsible for the proliferation and maintenance of GC precursors (61), was undetected upon GC differentiation. ATOH1 maintains cilia and SHH responsiveness in GC progenitors partly by promoting expression of the centriolar satellite protein CEP131 (10) and by synergizing with GLI2 in activating target genes (62). Thus, the reprogramming that impacted expression of IFT, PCM, and centriolar satellite genes coincided with the loss of ATOH1 during differentiation and decreased expression of SHH pathway components.

Termination of SHH signaling may also globally reduce translation. A direct target gene of SHH is the N-MYC gene, *Mycn*, a master transcriptional regulator of ribosome biogenesis (63). Both the frequency and level of *Mycn* expression decreased during the final stages of neurogenesis (*SI Appendix*, Fig. S6*A*). In addition, expression pattern clusters 1 and 4 (Fig. 1*C*) included many significant GO terms related to protein biogenesis. Reduction in translation could also impact cilia maintenance.

**Fig. 5.**
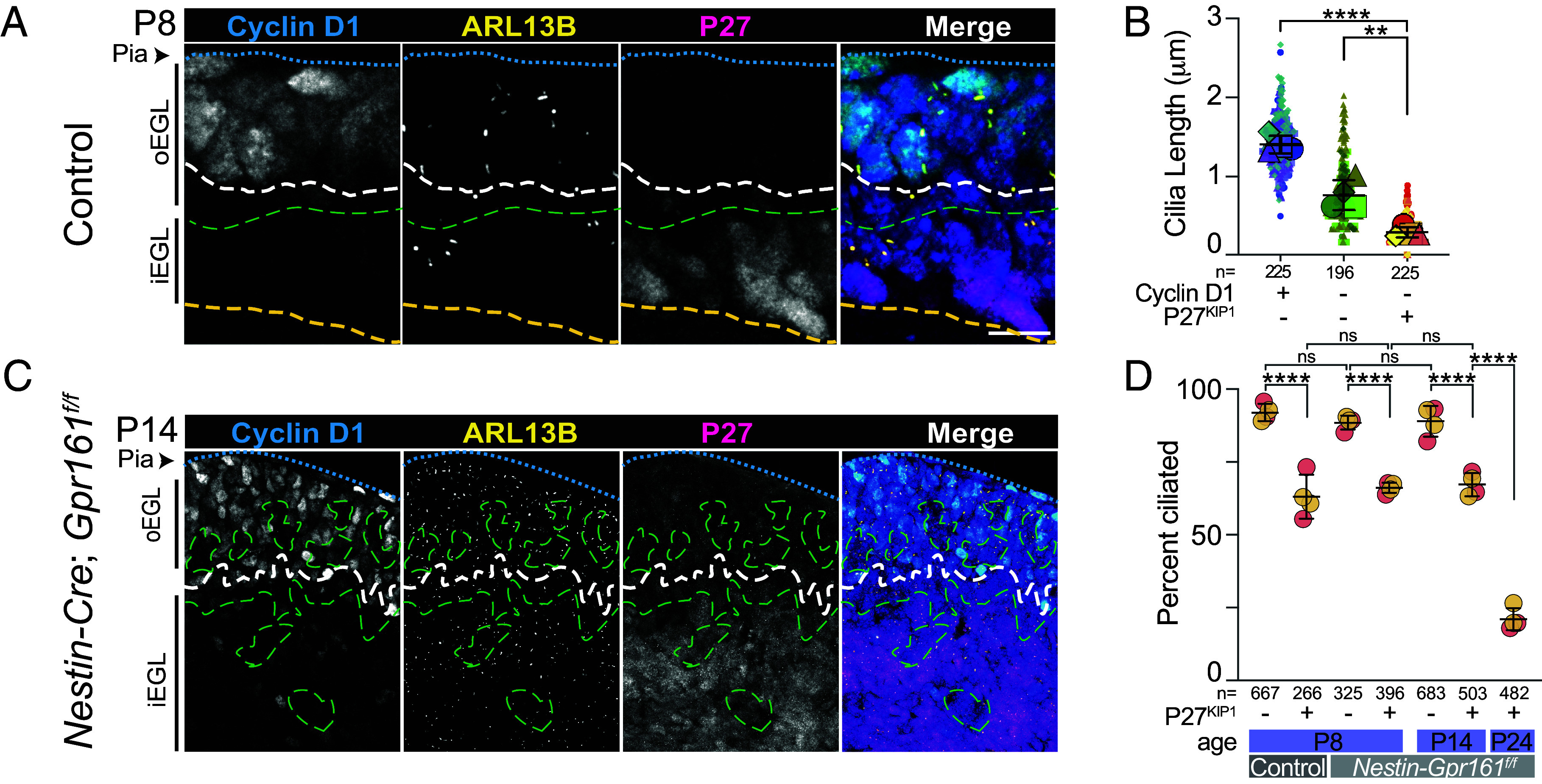
SHH signaling downregulation during GC differentiation coincided with ciliary loss. (*A*) Sagittal sections of P7 mice cerebella were stained with antibodies to the SHH pathway transcriptional target cyclin D1, the ciliary marker protein ARL13B and the GC differentiation marker P27^KIP1^ along with costaining with DAPI (blue) prior to imaging by widefield microscopy. The blue dashed line indicates the Pial surface, the white line is the lower boundary of cyclin D1 positive cells, the green line is the upper boundary of the P27^KIP1^ positive cells, and the yellow dashed line indicates the inside edge of EGL. Neither cyclin D1 nor P27^KIP1^ are detected in the cells between the white and green lines. (*B*) Cilia lengths were measured in three sections from each of three animals. Lengths were plotted based on the expression of cyclin D1 and P27^KIP1^ and the average cilium length for each animal is indicated as a large shape. Statistical analysis was preformed using multiple comparison ANOVA. (*C*) Sagittal sections of P14 *Nestin-Cre; Gpr161^f/f^* mice cerebella were stained as in (*C*). Persistent EGL in *Nestin-Cre; Gpr161^f/f^* mice was dysplastic. The blue dashed line indicates the Pial surface, the white line is roughly the lower boundary of cyclin D1 positive cells, and the green dashed line indicates the upper boundary of the P27^KIP1^ positive cells. Inside edge of EGL is not seen due to thickness of EGL (See *SI Appendix*, Fig. S6). Cells that stain with neither cyclin D1 nor P27^KIP1^ are dispersed throughout the EGL (dashed green circles). (*D*) Cilia frequency was measured in control and *Nestin-Cre; Gpr161^f/f^* mice (designated as *Nestin-Gpr161^f/f^*) EGL from 2 regions each from 2 mice/genotype at designated ages. Statistical analysis was preformed using multiple comparison ANOVA. Scale bar: (*A*) and (*C*), 10 μm.

To evaluate the relationship between ciliation and SHH signaling, we stained P7 cerebella sections with antibodies to ARL13B, P27^KIP1^, and the direct target of SHH regulation, cyclin D1 (encoded by the gene *Ccnd1*) (56). As expected, cyclin D1 was present in the ciliated GC progenitors of the outer EGL (Fig. 5*A*). We also found cells negative for both cyclin D1 and P27^KIP1^, positioned similar to the PCM1/P27^KIP1^ negative cells in Fig. 4*B*. High cyclin D1 levels during the cell cycle can drive G1-S progression in the absence of SHH signaling in daughter cells (64, 65). Therefore, cells lacking both cyclin D1 and P27^KIP1^ were likely completing a final cycle prior to differentiation (triggered by cyclin D1 levels from the previous cell cycle). GCs negative for both cyclin D1 and P27^KIP1^ had shorter cilia than the cyclin D1 positive cells (Fig. 5*B*). Cilia in P27^KIP1^ positive cells were even shorter (Fig. 5*B*).

We next altered SHH signaling and assessed cilia deconstruction in *Nestin-Cre; Gpr161^f/f^* mice that lack the SHH repressor, GPR161. *Nestin-Cre; Gpr161^f/f^* mice have excessive GC progenitor proliferation, persistent dysplastic EGL, and delayed neuronal maturation (66). The resulting EGL expansion is illustrated in *SI Appendix*, Fig. S6 *B* and *C*. Cilia frequency distribution in *Nestin-Cre; Gpr161^f/f^* mice at both P8 and P14 was comparable to WT P8 mice (Fig. 5 *C* and *D*). P27^KIP1^ positive GCs had shorter cilia than P27^KIP1^ negative progenitors (Fig. 5*C* and *SI Appendix*, Fig. S6 *B* and *D*), and GCs negative for both cyclin D1 and P27^KIP1^ had shorter cilia than the cyclin D1 positive progenitors (Fig. 5*C* and *SI Appendix*, Fig. S6*E*). Only P24 *Nestin-Cre; Gpr161^f/f^* mice exhibited decreased cilia frequency (Fig. 5*D* and *SI Appendix*, Fig. S6*B*). Thus, while SHH signaling promoted both proliferation and cilia maintenance in the absence of GPR161, differentiation and cilia deconstruction were not prevented, only delayed.

### Capping Proteins Prevented Cilia Elongation from Docked Centrioles.

**Fig. 6.**
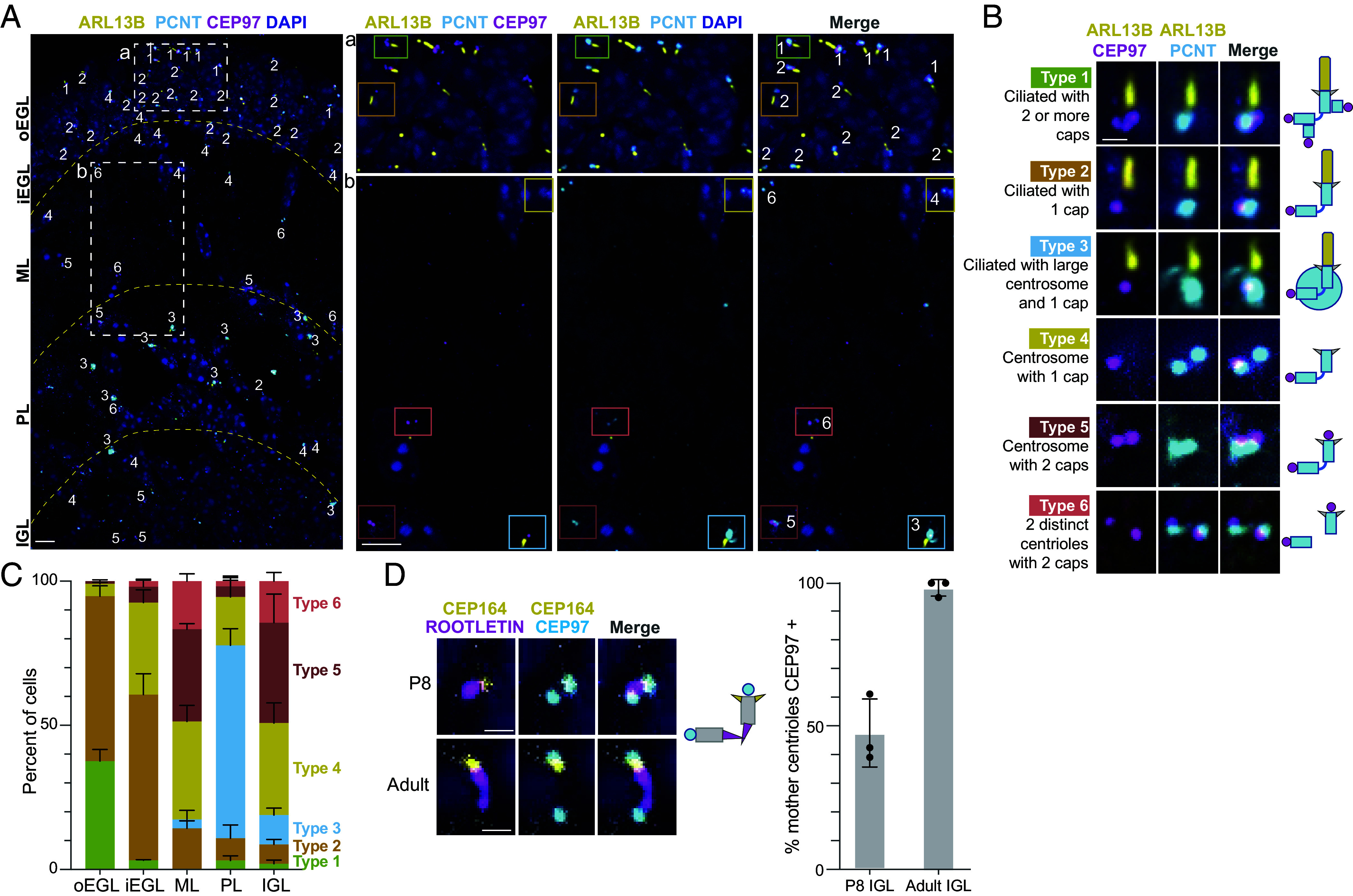
Centriole capping in mature GC neurons prevented regrowth of primary cilia. (*A*) Sagittal sections of P8 mice cerebella stained with indicated antibodies and costained with DAPI were imaged using spinning disk confocal microscopy. Dotted lines indicate boundaries between cerebellar layers. The boxed regions (*a*, *b*) are magnified and shown as channel subsets to the *Right*. Centrosomes were classified based on the presence or absence of a cilium, the area of the PCNT signal and the number of adjacent CEP97 puncta. (*B*) Representative images and an illustration of each class are shown. Each image corresponds to the square in A with coordinating color. (*C*) The distribution of centrosome type is graphed for each layer. 210 to 327 centrioles per animal were classified from three animals. (*D*) Sagittal sections from adult and P8 cerebellum were stained with antibodies against the distal appendage protein CEP164, distal centriole associated rootletin, the capping protein CEP97 and counterstained with DAPI before imaging as in (*A*). The percentage of centrosomes with two CEP97 puncta is graphed. 36 to 145 mother centrioles were assessed from each of three mice. Adult mice were aged P39/P50/P160. Scale bar: (*A*), 5 μm; (*B*) and (*D*), 1 μm.

Centriole docking at the plasma membrane can be an early step in cilia formation (67), yet cilia do not regrow after cilia deconstruction (12). To investigate whether the CEP97 and CP110 capping complex, which is removed from the distal mother centriole prior to ciliogenesis (68, 69), is recruited during GC neuronal maturation, we immunostained P8 cerebellar tissue with antibodies to CEP97, ARL13B, and PCNT (Fig. 6*A*). The capping complex associates with mature daughter centrioles (68, 70), so every centrosome had at least one CEP97 puncta. We scored six different configurations of cilia, centrosomes, and caps as shown in Fig. 6*B*. CEP97 was not associated with basal bodies and duplicating centrioles had two or three CEP97 puncta. Large bright PCNT staining located in the Purkinje cell layer on ciliated glia or Purkinje neurons were classified separately (*SI Appendix*, Fig. S3). The nonciliated centrioles were classified as having either one or two CEP97 puncta. A single PCNT puncta with two CEP97 puncta indicated that the mother and daughter centrioles were too close to resolve.

To evaluate changes in CEP97 binding, we quantified the distribution of the centriole classes across each layer (Fig. 6*C*). In the outer EGL 95 % of GCs had cilia and one, two, or three CEP97 puncta. In the inner EGL, ~60% of GCs had cilia and a single CEP97 puncta and nonciliated cells typically had a single CEP97 puncta. The fraction of nonciliated cells with two capped centrioles increased as differentiation progressed. In the IGL, centrioles with two CEP97 puncta were more frequent than cells with an uncapped centriole, suggesting that mother centriole capping occurs as differentiation progresses. To determine whether mother centrioles remained capped, we sectioned both P8 and adult cerebellar tissue and stained with antibodies to CEP97 and two markers: the distal appendage protein CEP164 and the protein rootletin, which associates with the centriole. Approximately half of the P8 IGL centrosomes had two CEP97 puncta and in the adult tissue 100% of the cells had two Cep97 puncta (Fig. 6*D*). These data indicate that in addition to cilia deconstruction, GC maturation likely involves centriole capping to prevent cilia reformation, despite mother centriole docking at the plasma membrane.

## Discussion

This study reveals molecular changes that occur as cilia deconstruct in vivo during GC neurogenesis. Transcripts for molecular triggers active in premitotic cilia resorption (18, 19) were reduced and instead we found a gradual decrease in factors required for cilia maintenance. Global changes in transcription and translation, initiated at least in part by downregulation of SHH signaling, coincided with reductions in cilia length. Therefore, we propose that cilia disassembly occurs because multiple components required to maintain cilia were downregulated. Moreover, CEP97, a component of the centriolar cap that prevents ciliogenesis, was recruited to mother centrioles, likely preventing cilia regrowth after cilia deconstruction.

To our knowledge, withdrawal of cilia maintenance has not previously been proposed as a developmentally programmed cilia disassembly mechanism. Organelle maintenance has been defined as “all processes in a cell that prevent a given property from deteriorating” (30). Considerations of cilia and centrosome maintenance have largely focused on organelle stability (71, 72) and aging-related phenotypic changes (73). IFT, centriolar satellites, and the PCM all facilitate recruitment and assembly of cilia components (30). Transition zone component transcripts were also diminished but protein loss could not be validated with immunostaining. A requirement for IFT in cilia maintenance has been demonstrated in multiple systems. *Chlamydomonas* flagella shorten upon inhibition of anterograde IFT in temperature-sensitive kinesin-II mutants (74, 75), and NIH3T3 cells lose cilia upon acute inhibition of kinesin-II (76). Interplay between centriolar satellites, the PCM, IFT, and other local components could be necessary for cilia maintenance. Centriolar satellite integrity requires centriole-independent mechanisms (77, 78), including phosphorylation of PCM1 (79) and CEP131 (80). PCM1 regulates IFT levels in and around cilia (81). The peribasal body recruitment of IFT is regulated by diffusion and capture by the basal body rather than by microtubular transport (82). Therefore, the loss of centriolar satellite and PCM proteins could undermine cilia maintenance through diminished IFT recruitment.

EM images of maturing and adult GC neurons show that unciliated mother centrioles dock at the plasma membrane (12). In most systems, centriole docking is followed by extension of a cilium from the cell surface. In cortical apical neural progenitors centrosome anchoring at the basolateral surface precedes cilia formation both following mitosis and during conversion of apical to basal neural progenitors (83, 84). Capping of docked, unciliated mother centrioles by the CP110/CEP97 complex has only been reported in cytotoxic T cells (85). Here, we showed that capping occurred following cilia deconstruction. Because centriole docking and capping were partial in the IGL during postnatal development and universal in adult tissue (12), we speculate that the processes of docking and capping are coordinated. Depletion of centriolar satellite proteins could facilitate recruitment of CP110 and CEP97, which were retained at the centrosome in mice and cultured cells lacking PCM1 (81).

The transcriptomic and immunostaining strategies each had limitations. The curated gene list was extensive, but not exhaustive, and several transcripts were not detected above the threshold of 5% of cells. In addition, many antibodies provided insufficient signal or excessive nonspecific signal in tissue. Transcriptional regulation is only one of several determinants that contribute to periciliary proteome remodeling. Decreased transcription of N-MYC and protein biogenesis genes could alter protein biosynthesis (63). Basal body and centriolar satellite components also locally coordinate with protein degradation machinery (86). In GC progenitors, the E3 ligase SIAH2 prevents cilia disassembly (9), whereas the centrosome localized E3 ligase HUWE1 promotes cilia disassembly by degradation of TTBK2, a kinase pivotal for ciliogenesis and cilia stabilization (87–89). Turnover of component proteins also facilitates centriole and basal body maintenance and remodeling (72, 90) and local regulation in inclusions, such as aggresomes, could be important (91). We predict that both global and local factors impact cilia/centrosome proteome maintenance.

Although the downregulation of IFT, PCM, and centriolar satellite genes was striking, several cilia and centrosome genes were transcribed at higher levels in differentiating and mature GC neurons including genes implicated in ciliogenesis: *Dvl3, Ehd3, Pacsin1, Rab11a, Rab11b,* and the Trapp II complex genes (92). The DAVID analyses also found the GO term “cilium assembly” associated with opposite trending clusters (clusters 1 and 7, and 2 and 5). Genes upregulated in differentiating and mature GC neurons could have functions separate from their roles in cilia including recovery of centriole-associated membranes, promoting mother centriole docking, or facilitating migration, differentiation, and maturation. For example, PACSIN1 has been implicated in formation of membrane tubules from ciliary vesicles (93), features that we reported during cilia deconstruction in differentiating GCs (12). RAB11 and the TRAP II complex contribute to cell polarization and intracellular trafficking in multiple contexts (94, 95). The contributions of individual ciliogenesis genes during GC neurogenesis will require further study.

Experiments testing the proposed cilia deconstruction mechanism will be challenging because current tools for in vivo manipulations are insufficient. Conventional single or multiple gene perturbations in situ may not be enough to disrupt cilium maintenance. For example, centriolar satellite loss in *Pcm1^Gt/Gt^* mice was unable to accelerate ciliary deconstruction. In contrast to *Chlamydomonas* and cultured cells, loss of individual IFT components from established cilia/flagella in *Trypanosoma* and *Drosophila* does not cause disassembly (31, 96). Key transcriptional regulators of IFT, satellite, and PCM components need to be identified and perturbed. However, we suspect redundancy in transcriptional regulation of IFT subunits because knockdown of the transcription factor *Rfx3* in the mouse cerebellar anlage shows limited developmental atrophy (28). ATOH1, a known regulator of GC proliferation, transcriptionally regulates a single satellite component CEP131 (10). The G1-S transition-regulating transcription factor *EZH2* has been implicated in downregulation of some cilia-related genes and decreased ciliation, also termed cilia deconstruction, in cycling metastatic melanoma cells (97). However, *EZH2* was expressed at higher levels in cycling GC progenitors than in differentiating and maturing GC neurons, and is distinct from postmitotic cilia disassembly described here.

A detailed understanding of developmentally programmed cilia disassembly might provide insights relevant to pathological cilia disassembly, for example in Parkinson’s disease (98). Investigating the proposed mechanisms that accomplish cilia removal and block cilia regrowth might also open unanticipated avenues to target medulloblastoma. GC tumor cells in both the SHH- and WNT-subtypes of medulloblastoma are ciliated (16, 17). Development of SHH-subtype medulloblastoma is preceded by formation of persistent proliferative nests of GCs (66). Additional therapeutic targets are needed because the use of Smoothened inhibitors, while initially promising, is restricted in young patients because it can cause skeletal growth retardation, premature growth plate closure, and drug resistance (99). It is not yet clear whether the ciliated tumor cells fail to deconstruct cilia, fail to cap cilia, or regrow cilia by reversal of cilia deconstruction and capping. Future studies will be required to determine how transcription and translation of IFT, PCM, and centriolar satellite components differ in ciliated GCs of the proliferative progenitor nests and in tumors. This knowledge could facilitate targeted therapeutic strategies to treat ciliated medulloblastoma subtypes.

## Materials and Methods

### scRNA-Seq Clustering and Analysis.

scRNA-seq gene expression matrices for mouse cerebellum P5 (GSM3318005), P7 (GSM3318006), and P14 (GSM3318007) developmental time points (26) were imported into Seurat v4.2.1 and combined [see notebooks (100)]. Briefly, single cells with fewer than 500 genes or greater than 10 percent mitochondrial RNA were excluded. The resulting expression data were normalized (“LogNormalize”, scale.factor = 10,000) and scaled (features = all genes). Cells were clustered by gene expression and UMAP projections using the first 20 principal components were created. Positive marker genes were identified whether expressed in a minimum of 25% of cells within a cluster with a minimum average log2 fold-change threshold of 0.25 (only.pos = T, min.pct = 0.25, logfc.threshold = 0.25).

### Gene Expression Pattern Clustering.

Hierarchical clustering was used to identify gene expression patterns. Using normalized scaled gene expression values from GC cell clusters, we calculated the mean expression within each GC cluster, then calculated the global mean expression per gene across all GCs and selected the top 5% [see R notebooks (100)] for subsequent analysis. *SI Appendix* contains additional details about the clustering and analysis.

### Mouse Handling, Genotyping, and Mouse Brain Processing.

All animal studies were approved in accordance with UT Southwestern Institutional Animal Care and Use Committee regulations and were conducted in accordance with NIH guidelines for the care and use of laboratory animals. *SI Appendix* contains additional details regarding mouse handling, genotyping, and sample processing.

### Immunofluorescence Staining, Light Microscopy, and Image Analysis.

Cerebella sections were thawed at room temperature and OCT was removed by immersion in PBS. Sections were blocked using 3% serum (donkey) in PBS with 0.3% Triton-X 100 for 30 min. Primary antibodies were diluted in blocking solution and incubated overnight at room temperature in a humid chamber. Sections were incubated with the indicated secondary antibodies for 2 h at room temperature. Stained tissues were mounted using Fluromount-G (Southern Biotech) and allowed to dry overnight before imaging. Details regarding primary antibodies, dilutions, imaging, and analysis using FIJI are described in *SI Appendix*.

### Graphing and Statistics.

All graphs and statistics were generated using Prism (GraphPad) or R. Superplots were generated by overlaying average values from each animal onto individual values. Statistical significance was determined in Prism using ordinary one-way ANOVA using multiple comparison analysis with Tukey correction. Population mean was assessed at a 95% CI and was considered significant at the following *P* values: 0.0332 (*), 0.0021 (**), 00002 (***), <0.0001 (****).

## Supplementary Material

Appendix 01 (PDF)

Dataset S01 (XLSX)

Dataset S02 (XLSX)

Dataset S03 (XLSX)

Dataset S04 (XLSX)

Dataset S05 (XLSX)

Dataset S06 (XLSX)

Dataset S07 (PDF)

## Data Availability

Code and tables data will be deposited in Figshare (100).
