# Supplementary material for: Permanent cilia loss during cerebellar granule cell neurogenesis involves withdrawal of cilia maintenance and centriole capping
Source: Proc Natl Acad Sci U S A. 2024 Dec 20;121(52):e2408083121. doi: 10.1073/pnas.2408083121 (PMC11670249; doi:10.1073/pnas.2408083121)
Supplement: Supplementary file 1 — Appendix 01 (PDF) [file pnas.2408083121.sapp.pdf]

Supporting Information for
Permanent cilia loss during cerebellar granule cell neurogenesis involves withdrawal of cilia maintenance and centriole capping

Sandii Constable[1,#], Carolyn M. Ott[2,#,*,] Andrew L. Lemire[2], Kevin White[1], Yu Xun[1], Amin Lim[1], Jennifer Lippincott-Schwartz[2,*], Saikat Mukhopadhyay[1,*].

* Correspondence: Carolyn Ott, Saikat Mukhopadhyay and Jennifer Lippincott-Schwartz

Email: ottc@janelia.hhmi.org, saikat.mukhopadhyay@utsouthwestern.edu and lippincottschwartzj@janelia.hhmi.org

This PDF file includes:

Supporting text
Figures S1 to S6
Tables S1 and S2
Legends for Datasets S1 to S7
Description of Software S1

Other supporting materials for this manuscript include the following:

Datasets S1 to S7
Software S1

SI Methods

Gene expression pattern clustering

Hierarchical clustering was used to identify gene expression patterns. Using normalized scaled gene expression values from GC cell clusters, we calculated the mean expression within each GC cluster, then calculated the global mean expression per gene across all GCs and selected the top 5% (see https://figshare.com/s/6c7884fbc44230023ebd for R notebooks) for subsequent analysis. The resulting dendrograms were cut with varying group sizes (k values). The gene expression groups were evaluated by inspecting groupings in the dendrograms and performing GO analysis of genes within each expression group using DAVID. A cutoff of k=10 was chosen for the combined list of the top 5% of expressed genes plus the curated genes, which performed similarly to a reduced set of genes (excluding curated genes) using a cutoff of k=9.

The genes included in each expression pattern cluster are included in Datasets S3 and S5. To identify gene families with similar gene expression patterns, we used the Database for Annotation, Visualization and Integrated Discovery (DAVID) (1). The GO terms enriched in each cluster are listed in Datasets S4 and S6. For analysis, we considered Biological Process (BP), Cellular Component (CC), or Molecular Function (MF) GO terms with statistical enrichment indicated by a Benjamini score < 0.005.

Curated list of centrosome and cilia genes

The curated gene list (Dataset S2) was compiled from both research articles and reviews. References used in compiling the list are provided in the SI Appendix, Table 1. This list included genes for diverse proteins shown to localize to and promote the function of cilia (SI Appendix, Table S2). Genes were classified as a cilium gene, a centrosome gene (a limited number were in both categories) or other biological process shown to influence cilia (e.g. polarity proteins, ubiquitin regulatory proteins). Genes were also further classified based on cilia or centrosome substructures where appropriate.

Mouse handling and genotyping

Mice were housed in standard cages that contained three to five mice per cage, with water and standard diet *ad libitum* and a 12 h light/dark cycle. Both male and female mice were analyzed in all experiments. CD1 mice were purchased from Jackson Labs and maintained under standard conditions. Mice mutant for *Pcm1* were a gift from Nicholas Katsanis, Northwestern University Feinberg School of Medicine (2). The *Gpr161* conditional allele targeting the third exon crossed has been described before (3) and is indexed in MGI as *Gpr161*$^{tm1.2Smuk}$, MGI: 6357710). *Nestin-Cre* mice (B6.Cg-Tg(Nes-cre)1Kln/J; Stock No. 003771) were obtained from Jackson Laboratory (Bar Harbor, ME). Mouse genotyping was performed as described before (2, 3) by PCR of genomic DNA obtained from ear biopsies or toe clips.

Mouse brain processing

Mice brain processing was performed as described in (4). Mice were procured at the appropriate age and fixed by trans-cardial perfusion using 4% paraformaldehyde (PFA) in PBS after appropriate anesthesia for their age (either isoflurane or cold exposure on ice) according to IACUC regulations. Brains were removed and further fixed in 4% PFA/PBS overnight at 4°C on a rotator, then immersed in 30% sucrose in PBS until brain sank to the bottom of the tube (~48hrs). Brains were cut in half in the sagittal direction and embedded cut face down in cryomolds using OCT embedding media (BioTek, USA) and frozen on dry ice until solid. Blocks were stored at -80°C until sectioning on a Leica Cryostat model CM1950 at 15-30 μm thickness. Sections were stored at -20°C or -80°C until staining.

Immunofluorescence staining

Immunostaining was performed using a similar protocol as described in (4). Cerebella sections were thawed at room temperature and OCT was removed by immersion in PBS. Sections were blocked using 3% serum (donkey) in PBS with 0.3% Triton-X 100 for 30 min. Primary antibodies were diluted in blocking solution and incubated overnight at room temperature in humid chamber. Sections were incubated with the indicated secondary antibodies for 2 h at room temperature.

Stained tissues were mounted using Fluromount-G (Southern Biotech) and allowed to dry overnight before imaging. Stained slides were imaged within 2-3 days and stored at 4°C (short term) or -20°C (long term). Primary antibodies: ARL13B (1:1000, UC Davis/NeuroMab #75-287), CDK5RAP2 (1:500, Bethyl #IHC-00063-T), CEP131 (1:500, Proteintech #25735-1-AP), CEP164 (1:500, Proteintech #22227-1-AP; 1:200, Proteintech #CL488-22227), CEP97 (1:200, Proteintech #22050-1-AP), cyclin D1 (1:500, ThermoScientific #RB-9104-S0), γTUB (1:500, Santa Cruz #sc-17787), $P27^{KIP1}$ (1:400, BD Biosciences) #610241), PCM1 (1:500, Bethyl #A301-149A-T), PCNT (1:500, BD Biosciences #611814), Rootletin (1:500, Merck-Millipore #ABN1686), SOX9 (1:500, Millipore #ABE571), TALPID3 (1:500, Proteintech 24421-1-AP). Rabbit polyclonal antibodies against IFT140 (1:500), IFT57 (1:500) and IFT88 (1:500) were kind gifts of Dr. Gregory Pazour, UMass Med School and required incubation with 0.05% SDS for 5 mins followed by several washes with PBS before blocking solution was applied. When two mouse antibodies were used, isotype specific secondary antibodies were used. To stain nuclei, Hoechst 33342 (10μg/ml) was included with the secondary antibodies or DAPI (1μg/ml) Sigma) was added to the final wash.

Light microscopy and Image Analysis
Imaging and analysis were performed similar to experiments described in (4). Images were acquired on a widefield microscope (AxioImager.Z1; ZEISS), confocal microscope (Zeiss LSM880) or a spinning disk confocal microscope (Nikon CSU-W1 SoRa). Images in the widefield microscope were acquired using a Plan Apochromat objective (40×/1.3 NA oil and 63×/1.4 NA oil) and sCMOS camera (PCO Edge; BioVision Technologies) controlled using Micro-Manager software (University of California, San Francisco) at room temperature. Images in the confocal microscope (Zeiss LSM880) were acquired using Plan Apochromat objective (63×/1.4 NA oil). Images in the spinning disk confocal microscope (Nikon CSU-W1 SoRa) were acquired using a Plan Apochromat objective (100×/1.45 NA oil), a sCMOS camera (Hamamatsu Orca-Fusion), and a Piezo z-drive for fast z-stack acquisition controlled using Nikon NIS-Elements software at room temperature. Between 10 and 30 z sections at 0.2 μm intervals were acquired.

Cilia length and number determination
Different cerebellum layers were identified using nuclei, and P27 markers. Cilia were traced manually and analyzed using FIJI (5). Cilia length was manually determined by tracing at zoom level 200-300% using the freehand draw tool, and measurement recorded using measure tool. Cilia already traced were permanently marked with draw tool to ensure unique cilia were measured when moving around the image. Each cerebellar layer was completed in entirety before moving on to the next. The number of cilia was determined by counting the number of cilia measured. The number of cells was determined by counting the total number of nuclei found in each section as stained by DAPI.

Centrosome area and intensity measurements
Centrosome area was determined on calibrated images by manually encircling the centrosome as determined by PCNT staining and measured using the Measurements tool in FIJI. Integrated density of centrosome-associated proteins was determined by following established protocols (6, 7). Briefly, centrosome intensity was measured within a defined area of a constant size (20px, approx. 1.5mm^2) encircling the centrosomal unit (centrosome +PCM) from the summed intensity projections of Z stacks. Intensity of the background in a near proximity of each centrosome was subtracted from the signal intensity. At least 40 centrosomes were measured for each condition/cell cycle stage.

Superplots were generated by overlaying average values from each animal onto individual values, as explained in (8).

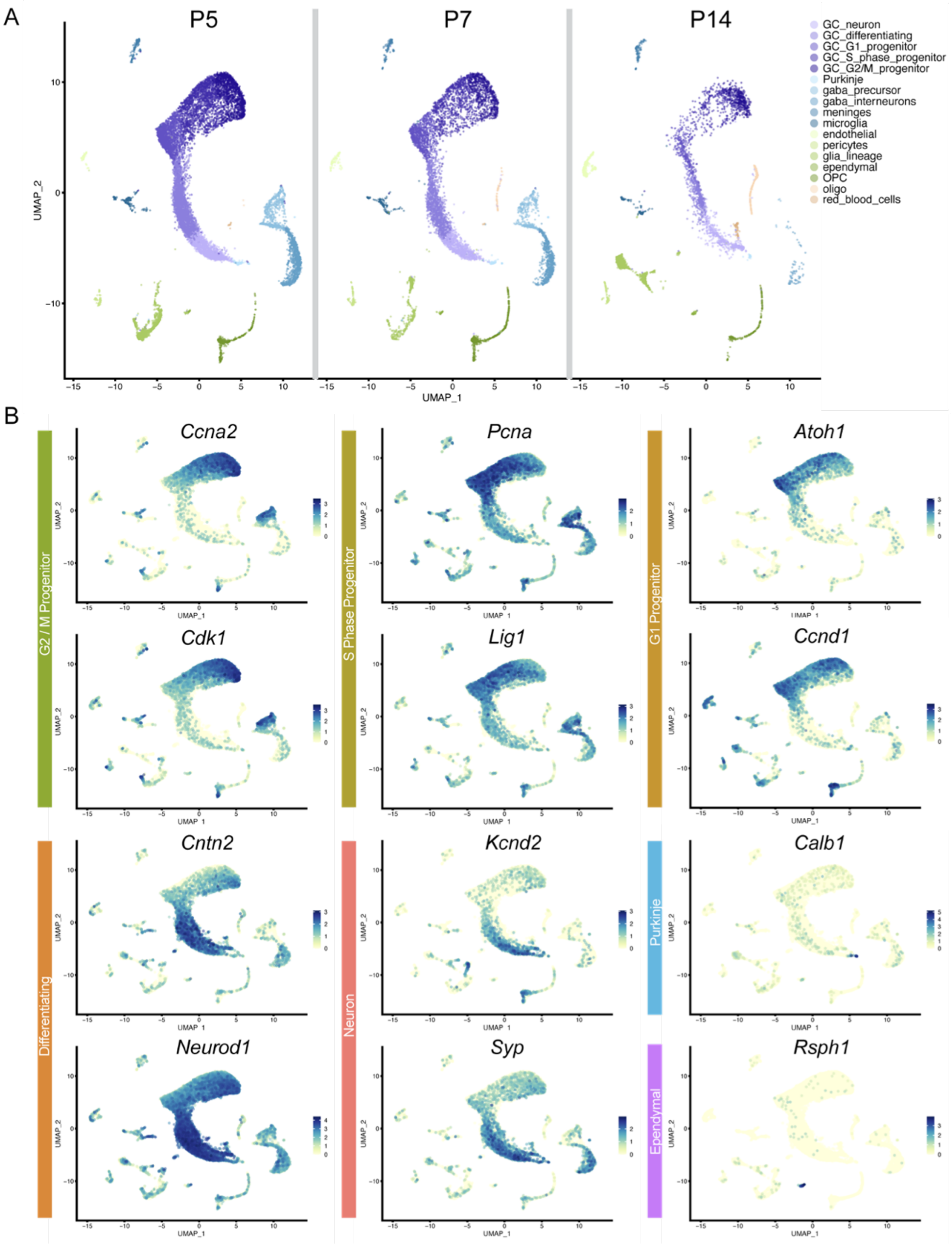

Fig. S1. Cell cluster expression pattern distributions.

(A) Cluster analysis was performed on the combined data from scRNA-seq from P5, P7 and P14 mice. Here the cluster assignments shown in Figure 1B were applied to the input data from each individual dataset. (B) The expression of the indicated genes across the combined dataset is shown. These genes are representative of genes with expression significantly enriched in the indicated cell cluster.

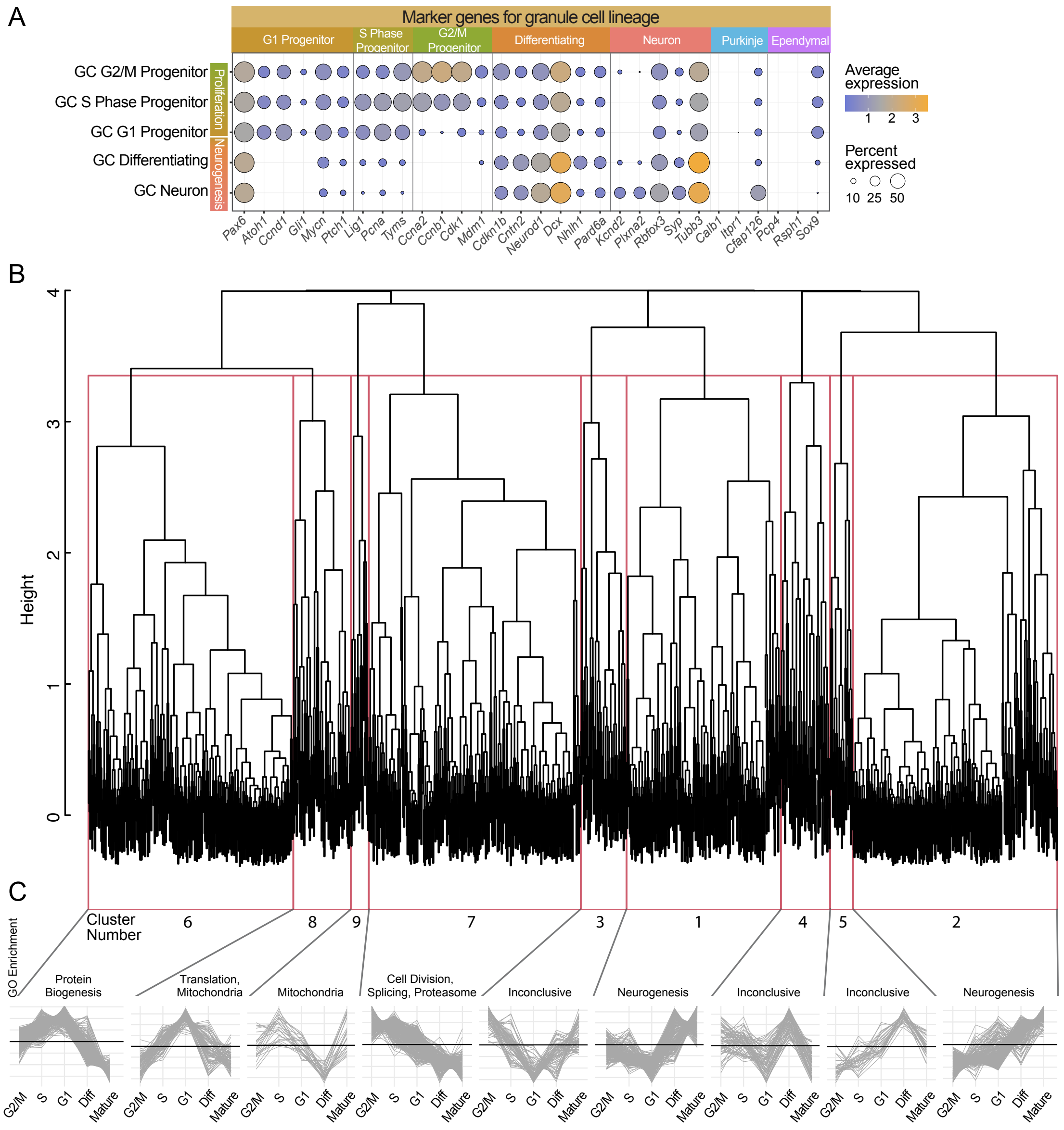

Fig. S2. Clustering of top 5% of GC genes by gene expression patterns.

(A) Expression of representative identifying genes expressed in the GC lineage were plotted. Purkinje neurons and ependymal cells are also included. (B) The top 5% of genes expressed in the granule cell lineage were identified and the expression patterns were normalized. Genes were clustered based on expression pattern similarities. We used a k=10 (cutoff value) represented by the top horizontal red line. (C) DAVID analysis of each cluster identified GO terms common to several genes in that cluster. A general term related to the common identifiers is listed above the graph of each expression pattern. Importantly, no cilia or centrosome GO terms were found in the DAVID analysis.

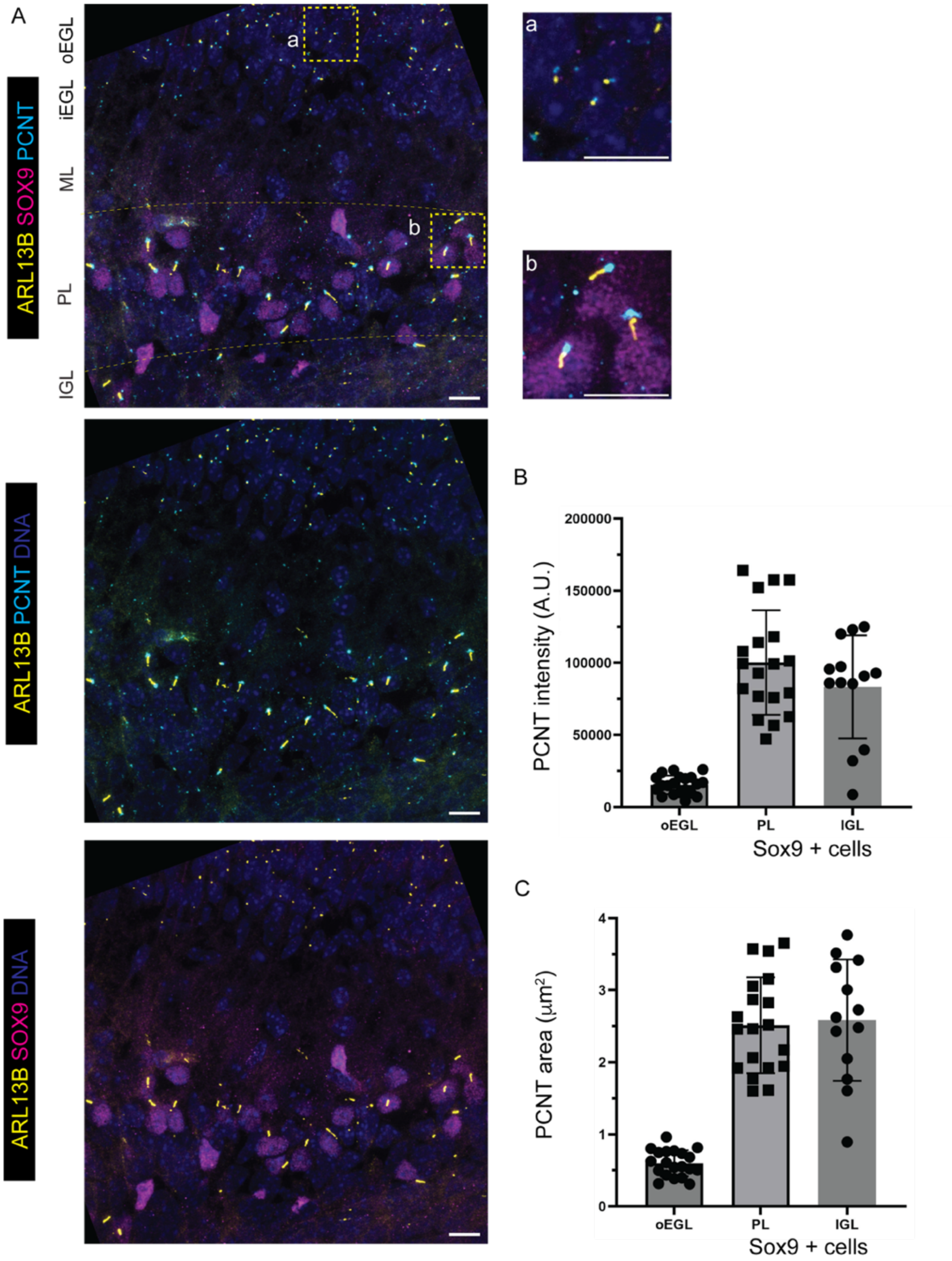

Fig. S3. PCNT can be detected in Purkinje cells and glial cells in the PCL and IGL.

(A) Sagittal sections of P8 mice cerebellum were stained with antibodies to the cilia marker ARL13B, the glial marker SOX9, the PCM marker PCNT and counterstained with DAPI. The yellow boxes indicate the location of the insets shown to the right. (B and C) The area and intensity of the fluorescence intensities of PCNT signal was measured in 3 sections from 1 animal. The intensity and area are plotted for the GCs in outer EGL, and the Sox9+ glia in the PL and IGL. Scale bar: (A) and (B), 10 μm.

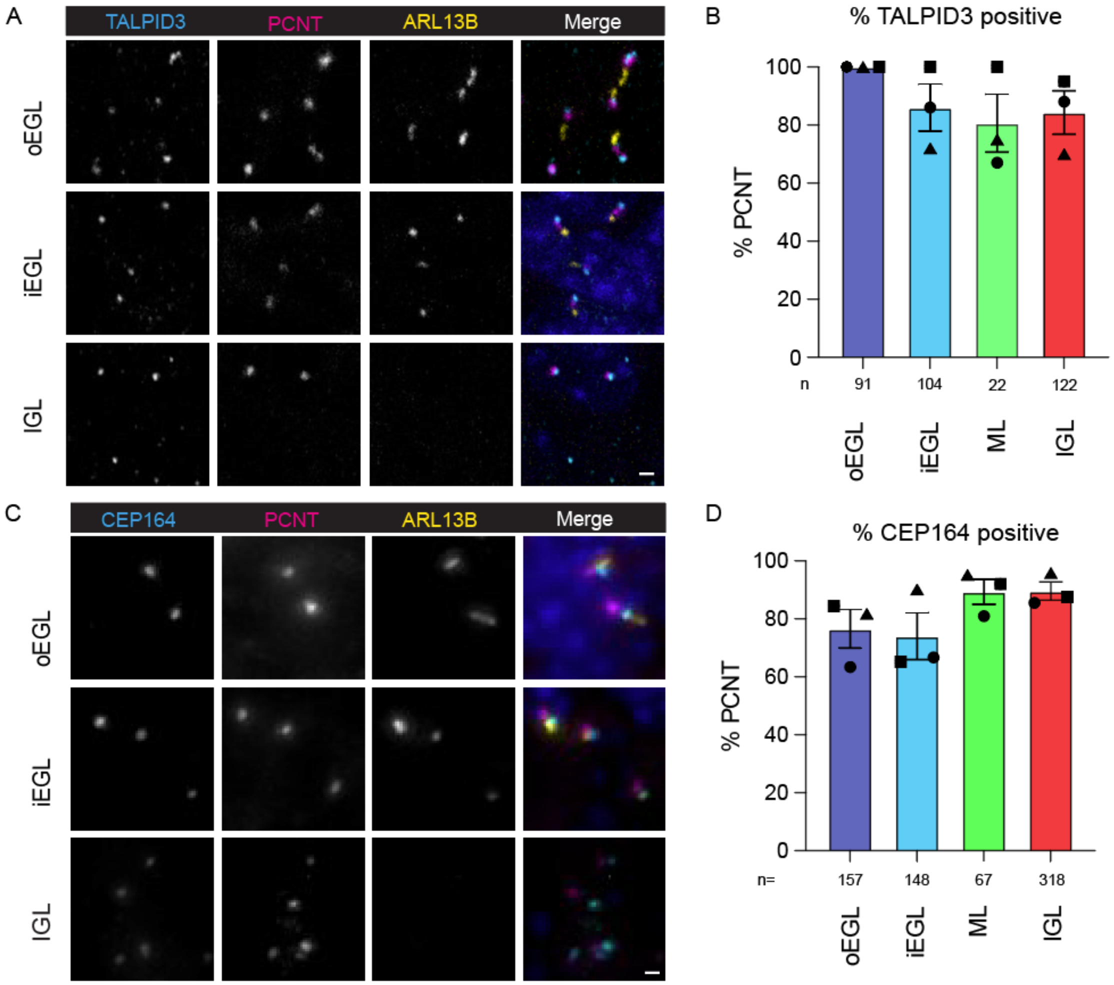

Fig. S4. Centriolar proteins are retained during GC maturation.

(A and C) Sagittal sections of P7 cerebellum were stained with antibodies to the centriole proteins CEP164 (A) or TALPID3 (C), the PCM marker PCNT, the cilia marker ARL13B, and counterstained with DAPI before imaging with confocal (A) or widefield (C) microscopy. Representative images from the outer and inner EGL and the IGL are shown. (B and D) The frequency of CEP164 (B) or TALPID3 (D) was adjacent to PCNT positive centrioles was measured in widefield images and is plotted as a percentage of total PCNT puncta in each layer. Both the distal appendage protein, CEP164, and the centriolar protein, TALPID3, were localized with PCNT in all GCs, irrespective of maturation. Quantifications was performed from one section/animal from 3 individual animals. Scale bar: 1 µm.

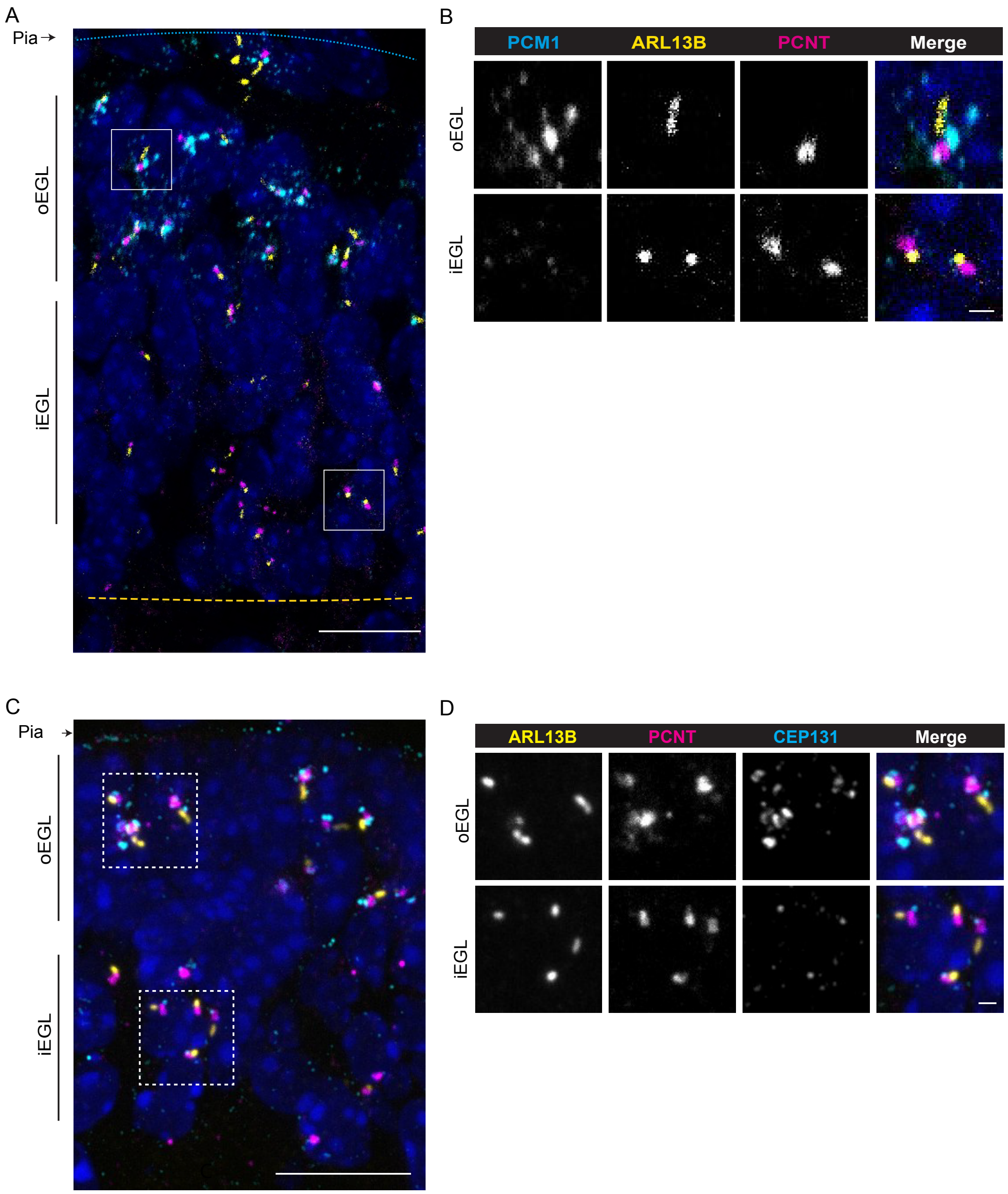

Fig. S5. Centriolar satellites are lost during GC maturation.

(A-B) Sagittal sections of P9 mice cerebella were stained with antibodies to PCM1 (centriolar satellite), ARL13B (cilium) and PCNT (PCM) and co-stained with DAPI (dark blue). The blue dashed line indicates the pial surface, and the yellow dashed line indicates the inside edge of EGL. The indicated regions of the outer EGL and inner EGL in A are magnified in B. (C-D) Sagittal section of P7 mice cerebellum were stained with antibodies to ARL13B, PCNT, the centriolar satellite protein CEP131, and counter-stained with DAPI before imaging using confocal microscopy. CEP131 detection was decreased in differentiating GCs. The indicated regions of the outer EGL and inner EGL in A are magnified in B. Scale bar: (A) and (B) 2.5 μm.

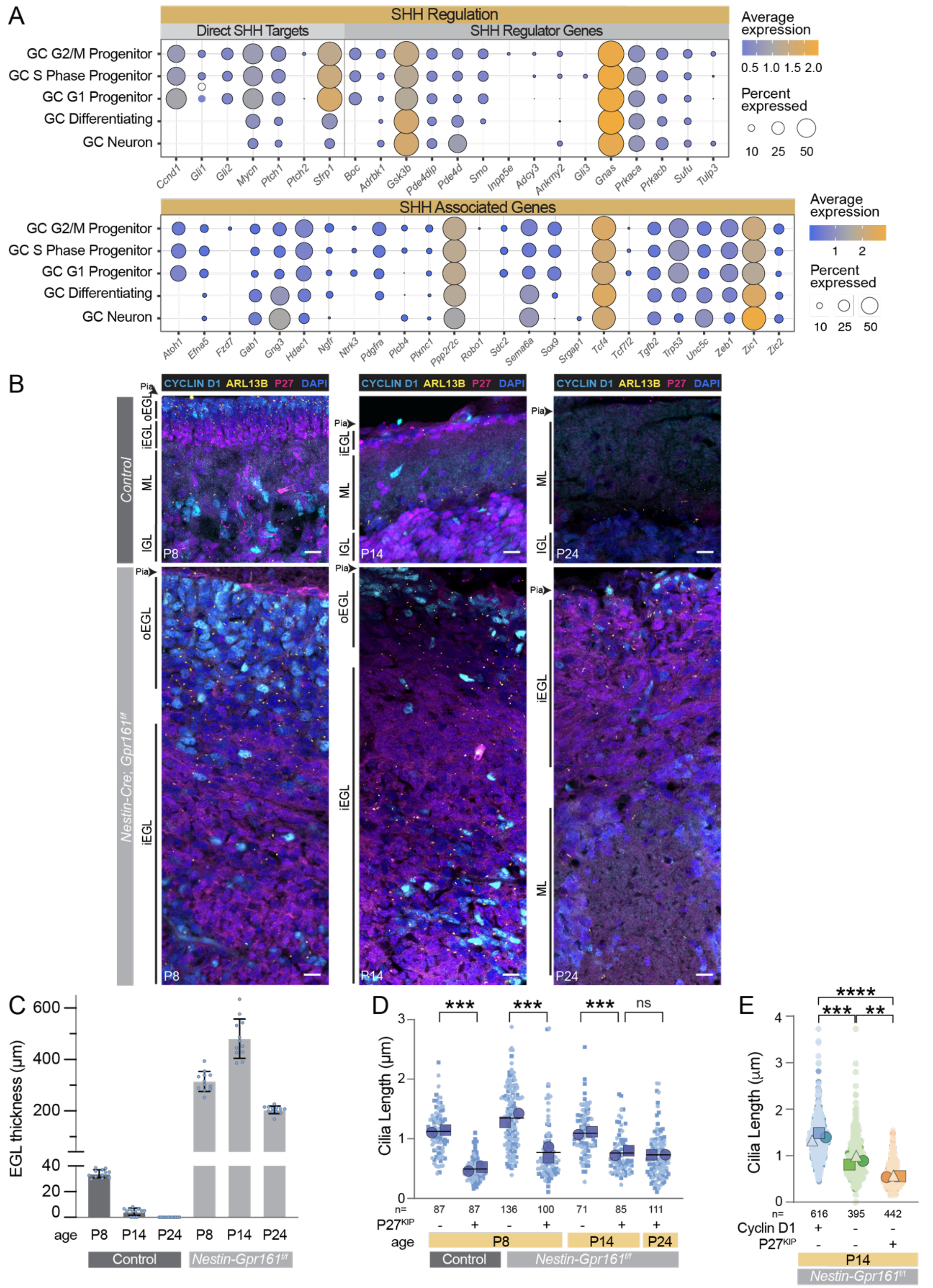

Fig. S6. SHH repression extended proliferation and delayed differentiation but did not prevent cilia deconstruction.

(A) Transcription of genes that are directly controlled by SHH pathway activity (top, left), genes coding for proteins that impact the SHH signaling cascade (top, right) and genes related to the SHH pathway (bottom) are graphed for each GC cluster. Transcript detection frequency is indicated by

dot size and dot color represents the average expression level. (B) Sagittal sections of P8, P14, P24 control littermate and *Nestin-Cre; Gpr161$^{f/f}$* mice cerebella were stained as in Figure 5(A). (C) Quantification of EGL thickness of posterior lobes 8-10 from 2 animals each/genotype shown at designated ages. (D) Quantification of cilia length from P27^{KIP1} negative and positive EGL GCs from 2 animals each/genotype shown at designated ages. Means from each animal are shown as a larger shape. Note lack of P27^{KIP1} positive GCs (outer EGL) in persistent EGL of *Nestin-Cre; Gpr161$^{f/f}$* mice (designated as *Nestin-Gpr161$^{f/f}$*). (E) Cilia lengths were measured from three P14 *Nestin-Cre; Gpr161$^{f/f}$* mice (*Nestin-Gpr161$^{f/f}$*) as in Fig 5(D). Means from each animal are shown as a larger shape. Statistical analysis was performed using multiple comparison ANOVA. Scale bars (B): 10 µm.

Table S1. Genes in the highest expressing GC genes (top 5%) with Centrosome and Cilia related GO terms.

Cluster label	Cluster # (discovery only)	Gene name	GO term	GO label	Protein name
Neurogenesis	1	*Calm3*	GO:0005813	centrosome	Calmodulin 3
Neurogenesis	1	*Dync1i2*	GO:0005813	centrosome	Dynein cytoplasmic 1 intermediate chain 2
Neurogenesis	1	*Bicd1*	GO:0005813	centrosome	BICD cargo adaptor 1
Neurogenesis	1	*Dctn3*	GO:0005813	centrosome	Dynactin 3
Neurogenesis	1	*Dynll2*	GO:0005813	centrosome	Dynein light chain LC8-type 2
Neurogenesis	1	*Cep170*	GO:0005813	centrosome	Centrosomal protein 170
			GO:0005814	centriole	
			GO:0120103	centriolar subdistal appendage	
Neurogenesis	1	*Fez1*	GO:0005813	centrosome	Fasciculation and elongation protein zeta 1
Neurogenesis	1	*Prkcb*	GO:0005813	centrosome	Protein kinase C, beta
Neurogenesis	1	*Atf4*	GO:0005813	centrosome	Activating transcription factor 4
Neurogenesis	1	*Mapre1*	GO:0005813	centrosome	Microtubule-associated protein, RP/EB family, member 1
			GO:0005815	microtubule organizing center	
			GO:0036064	ciliary basal body	
Neurogenesis	1	*Marcks*	GO:0005813	centrosome	Myristoylated alanine rich protein kinase C substrate
Neurogenesis	2	*Cdc42*	GO:0005813	centrosome	Cell division cycle 42
Neurogenesis	2	*Cct5*	GO:0005813	centrosome	Chaperonin containing TCP1 subunit 5
Neurogenesis	2	*Dctn2*	GO:0005813	centrosome	dynactin 2
Neurogenesis	2	*Crmp1*	GO:0005813	centrosome	Collapsin response mediator protein 1
Neurogenesis	2	*Ccna2*	GO:0005813	centrosome	Cyclin A2
Neurogenesis	2	*Atp6v0d1*	GO:0005813	centrosome	ATPase, H+ transporting, lysosomal V0 subunit D1
Neurogenesis	2	*Rab11a*	GO:0005813	centrosome	RAB11A, member RAS oncogene family
			GO:0005814	centriole	
			GO:0034451	centriolar satellite	
Neurogenesis	2	*Gsk3b*	GO:0005813	centrosome	Glycogen synthase kinase 3 beta
Neurogenesis	2	*Dynlrb1*	GO:0005813	centrosome	Dynein light chain roadblock-type 1

Neurogenesis	2	*Rbm39*	GO:0034451	centriolar satellite	RNA binding motif protein 39
Neurogenesis	2	*Akap9*	GO:0000242	pericentriolar material	A kinase anchor protein 9
			GO:0005813	centrosome	
			GO:0036064	ciliary basal body	
Neurogenesis	2	*Apc*	GO:0005813	centrosome	APC, WNT signaling pathway regulator
Neurogenesis	2	*Calm1*	GO:0005813	centrosome	Calmodulin 1
Neurogenesis	2	*Ndn*	GO:0005813	centrosome	Necdin, MAGE family member
Inconclusive	3	*Kif5b*	GO:0034451	centriolar satellite	Kinesin family member 5B
Inconclusive	3	*Pin1*	GO:0036064	ciliary basal body	Peptidyl-prolyl cis/trans isomerase, NIMA-interacting 1
Inconclusive	3	*Mzt1*	GO:0005813	centrosome	Mitotic spindle organizing protein 1
Inconclusive	3	*Ccdc88a*	GO:0005813	centrosome	Coiled coil domain containing 88A
			GO:0005814	centriole	
			GO:0036064	ciliary basal body	
Inconclusive	3	*Calm2*	GO:0005813	centrosome	Calmodulin 2
Inconclusive	4	*Rac1*	GO:0000242	pericentriolar material	Rac family small GTPase 1
Inconclusive	4	*Psmb4*	GO:0036064	ciliary basal body	Proteasome (prosome, macropain) subunit, beta type 4
Inconclusive	4	*Chd4*	GO:0005813	centrosome	Chromodomain helicase DNA binding protein 4
Inconclusive	5	*Txndc9*	GO:0005813	centrosome	Thioredoxin domain containing 9
Protein Biogenesis	6	*Ranbp1*	GO:0005813	centrosome	RAN binding protein 1
Protein Biogenesis	6	*Pcna*	GO:0005813	centrosome	Proliferating cell nuclear antigen
Protein Biogenesis	6	*Trp53*	GO:0005813	centrosome	Transformation related protein 53
Protein Biogenesis	6	*Ythdf2*	GO:0034451	centriolar satellite	YTH N6-methyladenosine RNA binding protein 2
Protein Biogenesis	6	*Rps7*	GO:0005813	centrosome	Ribosomal protein S7
Protein Biogenesis	6	*Npm1*	GO:0005813	centrosome	Nucleophosmin 1
Protein Biogenesis	6	*Psma1*	GO:0005813	centrosome	Proteasome subunit alpha 1
Protein Biogenesis	6	*Arl2bp*	GO:0005813	centrosome	ADP-ribosylation factor-like 2 binding protein
Protein Biogenesis	6	*Bccip*	GO:0005813	centrosome	BRCA2 and CDKN1A interacting protein
			GO:0005814	centriole	
Cell Division	7	*Top2a*	GO:0005814	centriole	Topoisomerase (DNA) II alpha

Cell Division	7	*Ddx3x*	GO:0005813	centrosome	DEAD box helicase 3, X-linked
Cell Division	7	*Birc5*	GO:0005814	centriole	Baculoviral IAP repeat-containing 5
Cell Division	7	*Ran*	GO:0005814	centriole	RAN, member RAS oncogene family
Cell Division	7	*Bcas2*	GO:0005813	centrosome	BCAS2 pre-mRNA processing factor
Cell Division	7	*Nudt21*	GO:0005813	centrosome	Nudix hydrolase 21
			GO:0034451	centriolar satellite	
Cell Division	7	*Ctnnb1*	GO:0005813	centrosome	Catenin beta 1
Cell Division	7	*Ccnb1*	GO:0005813	centrosome	Cyclin B1
Cell Division	7	*Ccnb2*	GO:0005813	centrosome	Cyclin B2
Cell Division	7	*Ccnd2*	GO:0005813	centrosome	Cyclin D2
Cell Division	7	*Cdk1*	GO:0005813	centrosome	Cyclin dependent kinase 1
Cell Division	7	*Mdh1*	GO:0005813	centrosome	Malate dehydrogenase 1, NAD (soluble)
Cell Division	7	*Ezr*	GO:0036064	ciliary basal body	Ezrin
Cell Division	7	*Tcp1*	GO:0000242	pericentriolar material	T-complex protein 1
			GO:0005813	centrosome	
			GO:0005815	microtubule organizing center	
Cell Division	7	*Hnrnpu*	GO:0005813	centrosome	Heterogeneous nuclear ribonucleoprotein U
Cell Division	7	*Cdc20*	GO:0005813	centrosome	Cell division cycle 20
Translation	8	*Apex1*	GO:0005813	centrosome	Apurinic/apyrimidinic endonuclease 1
Translation	8	*Ola1*	GO:0005813	centrosome	Obg-like ATPase 1
Translation	8	*Nme1*	GO:0005813	centrosome	NME/NM23 nucleoside diphosphate kinase 1
Translation	8	*Psmb5*	GO:0005813	centrosome	Proteasome (prosome, macropain) subunit, beta type 5
Translation	8	*Sfr1*	GO:0005813	centrosome	SWI5 dependent recombination repair 1
Neurogenesis	1	*Bbip1*	GO:0034464	BBSome	BBSome interacting protein 1
			GO:0060170	ciliary membrane	
			GO:0060271	cilium assembly	
Neurogenesis	1	*Gabarap*	GO:0005930	axoneme	Gamma-aminobutyric acid receptor associated protein
Neurogenesis	1	*Map1lc3b*	GO:0005930	axoneme	Microtubule-associated protein 1 light chain 3 beta

Neurogenesis	1	*Mapt*	GO:0005930	axoneme	Microtubule-associated protein tau
Neurogenesis	1	*Fnbp1l*	GO:0060271	cilium assembly	Formin binding protein 1-like
Neurogenesis	2	*Actr3*	GO:0060271	cilium assembly	ARP3 actin-related protein 3
Neurogenesis	2	*Clcn4*	GO:0097546	ciliary base	Chloride channel, voltage-sensitive 4
Neurogenesis	2	*Rab10*	GO:0005929	cilium	RAB10, member RAS oncogene family
Inconclusive	5	*Cd24a*	GO:0060170	ciliary membrane	CD24a antigen
Protein Biogenesis	6	*Arl2bp*	GO:0005929	cilium	ADP-ribosylation factor-like 2 binding protein
Protein Biogenesis	6	*Pkm*	GO:0005929	cilium	Pyruvate kinase, muscle
Cell Division	7	*Ccdc34*	GO:0005929	cilium	Coiled-coil domain containing 34
Neurogenesis	1	*Pafah1b1*	GO:0005813	centrosome	Platelet-activating factor acetylhydrolase, isoform 1b, subunit 1
			GO:0097730	non-motile cilium	
Neurogenesis	2	*Csnk1a1*	GO:0005813	centrosome	Casein kinase 1, alpha 1
			GO:0005929	cilium	
			GO:0036064	ciliary basal body	
Neurogenesis	2	*Atp6v1d*	GO:0005813	centrosome	ATPase, H+ transporting, lysosomal V1 subunit D
			GO:0005929	cilium	
			GO:0060271	cilium assembly	
Neurogenesis	2	*Atxn10*	GO:0005814	centriole	Ataxin 10
			GO:0036064	ciliary basal body	
			GO:0060271	cilium assembly	
Neurogenesis	2	*Prkar1a*	GO:0005813	centrosome	Protein kinase, cAMP dependent regulatory, type I, alpha
			GO:0005930	axoneme	
Inconclusive	3	*Dynll1*	GO:0005813	centrosome	Dynein light chain LC8-type 1
			GO:0035721	intraciliary retrograde transport	
Protein Biogenesis	6	*Cct4*	GO:0005813	centrosome	Chaperonin containing TCP1 subunit 4
			GO:0005929	cilium	
Protein Biogenesis	6	*Cct8*	GO:0005813	centrosome	Chaperonin containing TCP1 subunit 8
			GO:0005929	cilium	
Protein Biogenesis	6	*Ift27*	GO:0005813	centrosome	Intraflagellar transport 27
			GO:0005929	cilium	
			GO:0042073	intraciliary retrograde transport	
			GO:0060271	cilium assembly	
Cell Division	7	*Cetn3*	GO:0005813	centrosome	Centrin 3

			GO:0005814	centriole	
			GO:0005815	microtubule organizing center	
			GO:0035869	ciliary transition zone	
			GO:0036064	ciliary basal body	
Cell Division	7	*Pcm1*	GO:0000242	pericentriolar material	Pericentriolar material 1
			GO:0005813	centrosome	
			GO:0005814	centriole	
			GO:0034451	centriolar satellite	
			GO:0035869	ciliary transition zone	
			GO:0036064	ciliary basal body	
			GO:0060271	cilium assembly	
Cell Division	7	*Cenpf*	GO:0005813	centrosome	Centromere protein F
			GO:0005930	axoneme	
			GO:0036064	ciliary basal body	
			GO:0097539	ciliary transition fiber	
Cell Division	7	*Ssna1*	GO:0005813	centrosome	SS nuclear autoantigen 1
			GO:0005814	centriole	
			GO:0005929	cilium	
			GO:0005930	axoneme	
			GO:0036064	ciliary basal body	
			GO:0042073	intraciliary retrograde transport	
Translation	8	*Arl3*	GO:0005813	centrosome	ADP-ribosylation factor-like 3

Centrosome-related GO terms are shaded yellow and cilia-related GO terms are shaded green.

Table S2. References used to compile the curated list of genes coding for proteins that localize to and promote the function of centrosomes and cilia.

Category	Sub-category	References
Centrosome	Core centriole components	(9, 10)
	Pericentriolar matrix (PCM) proteins	(11–20)
	Additional centrosome localized proteins	(21–32)
	Distal and sub-distal appendage proteins	(9, 33–37)
	Distal centriole complex	(38)
	Centriolar satellites	(17, 39–41)
	Centriole cohesion	(35, 42–46)
	CP110-Cep97 cap regulation	(47–49)
Cilia	cilium assembly and disassembly	(26, 50–68)
	transition zone components	(9, 69, 70)
	Intraflagellar transport	(71)
	BBsome	(72)
	Additional cilia proteins	(73–94)
Other	Rab proteins	(95, 96)
	Microtubule motors	(73, 97–99)
	Tubulin modifying enzymes	(100–104)
	Vesicle trafficking	(65, 75, 89, 90, 93, 105)
	Polarity proteins	(106–109)
	SHH regulators	(110–114)
	SHH-associated	(115–120)
	Ubiquitination and Neddylation	(48, 58, 67, 121, 122)
	Proteins that function at or influence trafficking to immune synapses	(75, 105, 123–126)

The curated gene list (Dataset S2) was compiled from both research articles and reviews. Referenced papers may not be the first report of the gene/protein association with cilia or centrosomes. Although extensive, the literature references to genes included in the list may not be inclusive. Several references include proteins in several categories may be listed only once.

Dataset S1 (separate XLSX file). List of marker genes from cell clustering. Highly enriched genes (column B) are listed for each cluster (column A). Related to Figure 1B, 1C, and S1.

Dataset S2 (separate XLSX file). We determined the highest expressed genes (top 5%) in the GC clusters. For each gene, expression values were normalized across all GCs. The genes were then clustered based on the similarity of the pattern of gene expression. We separated the genes into 9 groups (k=9). Each tab of this table lists the genes and normalized expression values for each gene in each gene cluster. Related to Figure S2.

Dataset S3 (separate XLSX file). DAVID analysis of each expression group from top 5% of transcripts. The GO terms statistically significant enriched GO terms are listed for each group. The group label indicates a highly represented biological process within each group. Related to Figure S2.

Dataset S4 (separate XLSX file). Curated list of genes that localize to or regulate the cilia and centrosome. The *Ensembl* gene identifying numbers are listed in column A and the gene names are in column B. Genes shown in figures 1, 2, 3, 4 and 5 are also included in this dataset.

Dataset S5 (separate XLSX file). The top 5% of transcripts was combined with curated list of cilia/centrosome genes. The genes were then clustered based on the similarity of the pattern of normalized gene expression. We separated the genes into 10 groups (k=10). Each tab of this table lists the genes and normalized expression values for each gene in each gene cluster. Related to Figure 1D.

Dataset S6 (separate XLSX file). DAVID analysis of each expression group from the top 5% of transcripts combined with curated list of cilia/centrosome genes (k=10). Cilia or centrosome-related GO terms are shaded yellow. The group label indicates a highly represented biological process within each group. Related to Figure 1D.

Dataset S7 (separate PDF file). Dot plots show the expression of genes in the curated list of cilia/centrosome genes (detected in at least 5% of cells in that cluster) and then the top 5 % of genes expressed in GCs. Genes are listed in alphabetical order with each group. The fraction of cells in which each transcript was detected is represented by dot size (percent expressed). The dot color indicates the expression level in the cells where transcript was present (average expression). Genes shown in figures 1, 2, 3, 4 and 5 are also included in this dataset.

Software S1 (separate file). R notebooks containing code used for transcriptomic data analysis is available through https://figshare.com/s/6c7884fbc44230023ebd. Notebook I includes raw data processing and gene name conversion. Notebook II includes cell cluster identification and figures. Notebook III includes supplemental dotplot dataset and installation directions for R notebook environment.

References

1. B. T. Sherman, *et al.*, DAVID: a web server for functional enrichment analysis and functional annotation of gene lists (2021 update). *Nucleic Acids Res.* **50**, W216–W221 (2022).

2. T. O. Monroe, *et al.*, PCM1 is necessary for focal ciliary integrity and is a candidate for severe schizophrenia. *Nat. Commun.* **11**, 5903 (2020).

3. S. Hwang, *et al.*, The G protein-coupled receptor Gpr161 regulates forelimb formation, limb patterning and skeletal morphogenesis in a primary cilium-dependent manner. *Development* **145**, dev154054 (2017).

4. C. M. Ott, *et al.*, Permanent deconstruction of intracellular primary cilia in differentiating granule cell neurons. *J. Cell Biol.* **223**, e202404038 (2024).

5. J. Schindelin, *et al.*, Fiji: an open-source platform for biological-image analysis. *Nat. Methods* **9**, 676–682 (2012).

6. D. Keller, *et al.*, Mechanisms of HsSAS-6 assembly promoting centriole formation in human cells. *J. Cell Biol.* **204**, 697–712 (2014).

7. M. Bowler, *et al.*, High-resolution characterization of centriole distal appendage morphology and dynamics by correlative STORM and electron microscopy. *Nat Commun* **10**, 993 (2019).

8. S. J. Lord, K. B. Velle, R. D. Mullins, L. K. Fritz-Laylin, SuperPlots: Communicating reproducibility and variability in cell biology. *J Cell Biol* **219**, e202001064 (2020).

9. G. D. Gupta, *et al.*, A Dynamic Protein Interaction Landscape of the Human Centrosome-Cilium Interface. *Cell* **163**, 1484–1499 (2015).

10. A. Vasquez-Limeta, J. Loncarek, Human centrosome organization and function in interphase and mitosis. *Semin. Cell Dev. Biol.* **117**, 30–41 (2021).

11. J. B. Woodruff, O. Wueseke, A. A. Hyman, Pericentriolar material structure and dynamics. *Philosophical Transactions Royal Soc Lond Ser B Biological Sci* **369**, 20130459–20130459 (2014).

12. S. Lawo, M. Hasegan, G. D. Gupta, L. Pelletier, Subdiffraction imaging of centrosomes reveals higher-order organizational features of pericentriolar material. *Nat Cell Biol* **14**, 1148–1158 (2012).

13. J. A. Barrera, *et al.*, CDK5RAP2 Regulates Centriole Engagement and Cohesion in Mice. *Dev. Cell* **18**, 913–926 (2010).

14. K. F. Sonnen, *et al.*, 3D-structured illumination microscopy provides novel insight into architecture of human centrosomes. *Biol Open* **1**, 965–976 (2012).

15. J. Fu, D. M. Glover, Structured illumination of the interface between centriole and peri-centriolar material. *Open Biol* **2**, 120104–120104 (2012).

16. K. Watanabe, D. Takao, K. K. Ito, M. Takahashi, D. Kitagawa, The Cep57-pericentrin module organizes PCM expansion and centriole engagement. *Nat. Commun.* **10**, 931 (2019).

17. N. Oshimori, X. Li, M. Ohsugi, T. Yamamoto, Cep72 regulates the localization of key centrosomal proteins and proper bipolar spindle formation. *EMBO J.* **28**, 2066–2076 (2009).

18. M. Wieczorek, T.-L. Huang, L. Urnavicius, K.-C. Hsia, T. M. Kapoor, MZT Proteins Form Multi-Faceted Structural Modules in the γ-Tubulin Ring Complex. *Cell Rep.* **31**, 107791 (2020).

19. V. Mennella, *et al.*, Subdiffraction-resolution fluorescence microscopy reveals a domain of the centrosome critical for pericentriolar material organization. *Nat Cell Biol* **14**, 1159–1168 (2012).

20. J. Magescas, J. C. Zonka, J. L. Feldman, A two-step mechanism for the inactivation of microtubule organizing center function at the centrosome. *eLife* **8**, e47867 (2019).

21. S. W. Clark, D. I. Meyer, Centractin is an actin homologue associated with the centrosome. *Nature* **359**, 246–250 (1992).

22. Y. L. Lee, *et al.*, Cby1 promotes Ahi1 recruitment to a ring-shaped domain at the centriole-cilium interface and facilitates proper cilium formation and function. *Mol Biol Cell* **25**, 2919–2933 (2014).

23. I. V. Nechipurenko, *et al.*, A Conserved Role for Girdin in Basal Body Positioning and Ciliogenesis. *Dev Cell* **38**, 493–506 (2016).

24. A. D. Nardo, *et al.*, Phenotypic characterization of Cdkl5-knockdown neurons establishes elongated cilia as a functional assay for CDKL5 Deficiency Disorder. *Neurosci. Res.* **176**, 73–78 (2022).

25. I. Barbiero, *et al.*, CDKL5 localizes at the centrosome and midbody and is required for faithful cell division. *Sci. Rep.* **7**, 6228 (2017).

26. Y. E. Greer, *et al.*, Casein kinase 1δ functions at the centrosome and Golgi to promote ciliogenesis. *Mol. Biol. Cell* **25**, 1629–1640 (2014).

27. I. Cervenka, *et al.*, Dishevelled is a NEK2 kinase substrate controlling dynamics of centrosomal linker proteins. *Proc. Natl. Acad. Sci.* **113**, 9304–9309 (2016).

28. S. Yoshida, *et al.*, The novel ciliogenesis regulator DYRK2 governs Hedgehog signaling during mouse embryogenesis. *eLife* **9**, e57381 (2020).

29. X. Zhao, *et al.*, Fidgetin-like 1 is a ciliogenesis-inhibitory centrosome protein. *Cell Cycle* **15**, 2367–2375 (2016).

30. J. Busselez, *et al.*, Cryo-Electron Tomography and Proteomics studies of centrosomes from differentiated quiescent thymocytes. *Sci. Rep.* **9**, 7187 (2019).

31. B. R. Mardin, *et al.*, Components of the Hippo pathway cooperate with Nek2 kinase to regulate centrosome disjunction. *Nat. Cell Biol.* **12**, 1166–1176 (2010).

32. I. Peset, I. Vernos, The TACC proteins: TACC-ling microtubule dynamics and centrosome function. *Trends Cell Biol.* **18**, 379–388 (2008).

33. R. Uzbekov, I. Alieva, Who are you, subdistal appendages of centriole? *Open Biol* **8**, 180062 (2018).

34. N. Huang, *et al.*, Hierarchical assembly of centriole subdistal appendages via centrosome binding proteins CCDC120 and CCDC68. *Nat. Commun.* **8**, 15057 (2017).

35. V. Pizon, *et al.*, hVFL3/CCDC61 is a component of mother centriole subdistal appendages required for centrosome cohesion and positioning. *Biol. Cell* **112**, 22–37 (2020).

36. A. Kodani, M. S. Sirerol-Piquer, A. Seol, J. M. Garcia-Verdugo, J. F. Reiter, Kif3a interacts with Dynactin subunit p150Glued to organize centriole subdistal appendages. *EMBO J.* **32**, 597–607 (2013).

37. P. Chang, T. H. Giddings, M. Winey, T. Stearns, ε-Tubulin is required for centriole duplication and microtubule organization. *Nat. Cell Biol.* **5**, 71–76 (2003).

38. D. Kumar, *et al.*, A ciliopathy complex builds distal appendages to initiate ciliogenesis. *J Cell Biol* **220**, e202011133 (2021).

39. V. Quarantotti, *et al.*, Centriolar satellites are acentriolar assemblies of centrosomal proteins. *EMBO J.* **38**, e101082 (2019).

40. S. L. Prosser, L. Pelletier, Centriolar satellite biogenesis and function in vertebrate cells. *J. Cell Sci.* **133**, jcs239566 (2020).

41. F. F. Kersten, *et al.*, The mitotic spindle protein SPAG5/Astrin connects to the Usher protein network postmitotically. *Cilia* **1**, 2 (2012).

42. T. Hardy, *et al.*, Multisite phosphorylation of C-Nap1 releases it from Cep135 to trigger centrosome disjunction. *J. Cell Sci.* **127**, 2493–2506 (2014).

43. D. Hossain, S. Y.-P. Shih, X. Xiao, J. White, W. Y. Tsang, Cep44 functions in centrosome cohesion by stabilizing rootletin. *J. Cell Sci.* **133**, jcs239616 (2020).

44. K. K. Ito, *et al.*, Cep57 and Cep57L1 maintain centriole engagement in interphase to ensure centriole duplication cycle. *J. Cell Biol.* **220**, e202005153 (2021).

45. R. He, *et al.*, LRRC45 Is a Centrosome Linker Component Required for Centrosome Cohesion. *Cell Rep.* **4**, 1100–1107 (2013).

46. N. Huang, *et al.*, M-Phase Phosphoprotein 9 regulates ciliogenesis by modulating CP110-CEP97 complex localization at the mother centriole. *Nat Commun* **9**, 4511 (2018).

47. Q. Xu, *et al.*, Phosphatidylinositol phosphate kinase PIPKIγ and phosphatase INPP5E coordinate initiation of ciliogenesis. *Nat Commun* **7**, 10777 (2016).

48. A. B. Gonçalves, *et al.*, CEP78 functions downstream of CEP350 to control biogenesis of primary cilia by negatively regulating CP110 levels. *eLife* **10**, e63731 (2021).

49. T. Kobayashi, W. Y. Tsang, J. Li, W. Lane, B. D. Dynlacht, Centriolar Kinesin Kif24 Interacts with CP110 to Remodel Microtubules and Regulate Ciliogenesis. *Cell* **145**, 914–925 (2011).

50. L. Wang, B. D. Dynlacht, The regulation of cilium assembly and disassembly in development and disease. *Development* **145**, dev151407 (2018).

51. I. Sánchez, B. D. Dynlacht, Cilium assembly and disassembly. *Nat Cell Biol* **18**, 711–717 (2016).

52. J. J. Malicki, C. A. Johnson, The Cilium: Cellular Antenna and Central Processing Unit. *Trends Cell Biol.* **27**, 126–140 (2017).

53. S. Shakya, C. J. Westlake, Recent advances in understanding assembly of the primary cilium membrane. *Fac Rev* **10**, 16 (2021).

54. E. Gabriel, *et al.*, CPAP promotes timely cilium disassembly to maintain neural progenitor pool. *EMBO J.* **35**, 803–819 (2016).

55. W. Ding, Q. Wu, L. Sun, N. C. Pan, X. Wang, Cenpj Regulates Cilia Disassembly and Neurogenesis in the Developing Mouse Cortex. *J. Neurosci.* **39**, 1994–2010 (2019).

56. T. Miyamoto, *et al.*, The Microtubule-Depolymerizing Activity of a Mitotic Kinesin Protein KIF2A Drives Primary Cilia Disassembly Coupled with Cell Proliferation. *Cell Rep.* **10**, 664–673 (2015).

57. K. H. Lee, *et al.*, Identification of a novel Wnt5a–CK1ε–Dvl2–Plk1-mediated primary cilia disassembly pathway. *EMBO J.* **31**, 3104–3117 (2012).

58. T. Ong, N. Trivedi, R. Wakefield, S. Frase, D. J. Solecki, Siah2 integrates mitogenic and extracellular matrix signals linking neuronal progenitor ciliogenesis with germinal zone occupancy. *Nat Commun* **11**, 5312 (2020).

59. S. Majumder, H. A. Fisk, VDAC3 and Mps1 negatively regulate ciliogenesis. *Cell Cycle* **12**, 849–858 (2013).

60. C. E. Jewett, *et al.*, RAB19 Directs Cortical Remodeling and Membrane Growth for Primary Ciliogenesis. *Dev Cell* **56**, 325-340.e8 (2021).

61. C. J. Westlake, *et al.*, Primary cilia membrane assembly is initiated by Rab11 and transport protein particle II (TRAPPII) complex-dependent trafficking of Rabin8 to the centrosome. *Proc National Acad Sci* **108**, 2759–2764 (2011).

62. S. C. Goetz, K. F. L. Jr., K. V. Anderson, The Spinocerebellar Ataxia-Associated Gene Tau Tubulin Kinase 2 Controls the Initiation of Ciliogenesis. *Cell* **151**, 847–858 (2012).

63. S. Xie, T. Farmer, N. Naslavsky, S. Caplan, MICAL-L1 coordinates ciliogenesis by recruiting EHD1 to the primary cilium. *J. Cell Sci.* **132**, jcs233973 (2019).

64. T. J. Park, B. J. Mitchell, P. B. Abitua, C. Kintner, J. B. Wallingford, Dishevelled controls apical docking and planar polarization of basal bodies in ciliated epithelial cells. *Nat. Genet.* **40**, 871–879 (2008).

65. M. Steger, *et al.*, Systematic proteomic analysis of LRRK2-mediated Rab GTPase phosphorylation establishes a connection to ciliogenesis. *Elife* **6**, e80705 (2017).

66. C. Chen, *et al.*, Ciliopathy protein HYLS1 coordinates the biogenesis and signaling of primary cilia by activating the ciliary lipid kinase PIPKIγ. *Sci. Adv.* **7**, eabe3401 (2021).

67. E. Senatore, *et al.*, The TBC1D31/praja2 complex controls primary ciliogenesis through PKA-directed OFD1 ubiquitylation. *EMBO J.* **40**, e106503 (2021).

68. E. N. Pugacheva, S. A. Jablonski, T. R. Hartman, E. P. Henske, E. A. Golemis, HEF1-Dependent Aurora A Activation Induces Disassembly of the Primary Cilium. *Cell* **129**, 1351–1363 (2007).

69. A. Li, *et al.*, Ciliary transition zone activation of phosphorylated Tctex-1 controls ciliary resorption, S-phase entry and fate of neural progenitors. *Nat. Cell Biol.* **13**, 402–411 (2011).

70. C. Li, *et al.*, MKS5 and CEP290 Dependent Assembly Pathway of the Ciliary Transition Zone. *Plos Biol* **14**, e1002416 (2016).

71. L. B. Pedersen, J. L. Rosenbaum, Intraflagellar transport (IFT) role in ciliary assembly, resorption and signalling. *Curr Top Dev Biol* **85**, 23–61 (2008).

72. X. Tian, H. Zhao, J. Zhou, Organization, functions, and mechanisms of the BBSome in development, ciliopathies, and beyond. *eLife* **12**, e87623 (2023).

73. R. Novas, *et al.*, Kinesin 1 regulates cilia length through an interaction with the Bardet-Biedl syndrome related protein CCDC28B. *Sci Rep-uk* **8**, 3019 (2018).

74. E. Reales, *et al.*, The MAL protein is crucial for proper membrane condensation at the ciliary base, which is required for primary cilium elongation. *J. Cell Sci.* **128**, 2261–2270 (2015).

75. C. M. Szalinski, A. Labilloy, J. R. Bruns, O. A. Weisz, VAMP7 Modulates Ciliary Biogenesis in Kidney Cells. *PLoS ONE* **9**, e86425 (2014).

76. L. Sang, *et al.*, Mapping the NPHP-JBTS-MKS protein network reveals ciliopathy disease genes and pathways. *Cell* **145**, 513–528 (2011).

77. T. Noguchi, K. Nakamura, Y. Satoda, Y. Katoh, K. Nakayama, CCRK/CDK20 regulates ciliary retrograde protein trafficking via interacting with BROMI/TBC1D32. *PLoS ONE* **16**, e0258497 (2021).

78. T. Kanie, *et al.*, The CEP19-RABL2 GTPase Complex Binds IFT-B to Initiate Intraflagellar Transport at the Ciliary Base. *Dev. Cell* **42**, 22-36.e12 (2017).

79. P. N. Adler, J. B. Wallingford, From Planar Cell Polarity to Ciliogenesis and Back: The Curious Tale of the PPE and CPLANE proteins. *Trends Cell Biol.* **27**, 379–390 (2017).

80. S. Kuhns, *et al.*, Rab35 controls cilium length, function and membrane composition. *EMBO Rep.* **20**, e47625 (2019).

81. G. V. Pusapati, *et al.*, EFCAB7 and IQCE Regulate Hedgehog Signaling by Tethering the EVC-EVC2 Complex to the Base of Primary Cilia. *Dev. Cell* **28**, 483–496 (2014).

82. E. Petsouki, *et al.*, FBW7 couples structural integrity with functional output of primary cilia. *Commun. Biol.* **4**, 1066 (2021).

83. A. Gerondopoulos, *et al.*, Planar Cell Polarity Effector Proteins Inturned and Fuzzy Form a Rab23 GEF Complex. *Curr. Biol.* **29**, 3323-3330.e8 (2019).

84. M. Zhong, *et al.*, Tumor Suppressor Folliculin Regulates mTORC1 through Primary Cilia. *J. Biol. Chem.* **291**, 11689–11697 (2017).

85. R. Ghossoub, *et al.*, Septins 2, 7 and 9 and MAP4 colocalize along the axoneme in the primary cilium and control ciliary length. *J. Cell Sci.* **126**, 2583–2594 (2013).

86. I. Franco, *et al.*, Phosphoinositide 3-Kinase-C2α Regulates Polycystin-2 Ciliary Entry and Protects against Kidney Cyst Formation. *J. Am. Soc. Nephrol.* **27**, 1135–1144 (2016).

87. J. R. Schaub, T. Stearns, The Rilp-like proteins Rilpl1 and Rilpl2 regulate ciliary membrane content. *Mol. Biol. Cell* **24**, 453–464 (2013).

88. N. Schwarz, *et al.*, Arl3 and RP2 regulate the trafficking of ciliary tip kinesins. *Hum. Mol. Genet.* **26**, 3451–3451 (2017).

89. S. H. Low, *et al.*, Targeting of SNAP-23 and SNAP-25 in Polarized Epithelial Cells. *J Biol Chem* **273**, 3422–3430 (1998).

90. Y. Chen, *et al.*, A SNX10/V-ATPase pathway regulates ciliogenesis in vitro and in vivo. *Cell Res.* **22**, 333–345 (2012).

91. A. Eblimit, *et al.*, Spata7 is a retinal ciliopathy gene critical for correct RPGRIP1 localization and protein trafficking in the retina. *Hum. Mol. Genet.* **24**, 1584–1601 (2015).

92. C. K. Lai, *et al.*, Functional characterization of putative cilia genes by high-content analysis. *Mol Biol Cell* **22**, 1104–1119 (2011).

93. J. Mazelova, N. Ransom, L. Astuto-Gribble, M. C. Wilson, D. Deretic, Syntaxin 3 and SNAP-25 pairing, regulated by omega-3 docosahexaenoic acid, controls the delivery of rhodopsin for the biogenesis of cilia-derived sensory organelles, the rod outer segments. *J. Cell Sci.* **122**, 2003–2013 (2009).

94. H. W. Ko, *et al.*, Broad-Minded Links Cell Cycle-Related Kinase to Cilia Assembly and Hedgehog Signal Transduction. *Dev. Cell* **18**, 237–247 (2010).

95. O. E. Blacque, N. Scheidel, S. Kuhns, Rab GTPases in cilium formation and function. *Small GTPases* **9**, 76–94 (2018).

96. S. Chiba, Y. Amagai, Y. Homma, M. Fukuda, K. Mizuno, NDR2-mediated Rabin8 phosphorylation is crucial for ciliogenesis by switching binding specificity from phosphatidylserine to Sec15. *EMBO J.* **32**, 874–885 (2013).

97. H. Ishikawa, J. Thompson, J. R. Y. III, W. F. Marshall, Proteomic Analysis of Mammalian Primary Cilia. *Curr Biol* **22**, 414–419 (2012).

98. S. P. Taylor, *et al.*, Mutations in DYNC2LI1 disrupt cilia function and cause short rib polydactyly syndrome. *Nat. Commun.* **6**, 7092 (2015).

99. D. Wang, *et al.*, Motility and microtubule depolymerization mechanisms of the Kinesin-8 motor, KIF19A. *eLife* **5**, e18101 (2016).

100. S. Nicot, *et al.*, A family of carboxypeptidases catalyzing α- and β-tubulin tail processing and deglutamylation. *Sci. Adv.* **9**, eadi7838 (2023).

101. W.-T. Yang, *et al.*, The Emerging Roles of Axonemal Glutamylation in Regulation of Cilia Architecture and Functions. *Front. Cell Dev. Biol.* **9**, 622302 (2021).

102. J. Ran, Y. Yang, D. Li, M. Liu, J. Zhou, Deacetylation of α-tubulin and cortactin is required for HDAC6 to trigger ciliary disassembly. *Sci. Rep.* **5**, 12917 (2015).

103. T. Shida, J. G. Cueva, Z. Xu, M. B. Goodman, M. V. Nachury, The major α-tubulin K40 acetyltransferase αTAT1 promotes rapid ciliogenesis and efficient mechanosensation. *Proc. Natl. Acad. Sci.* **107**, 21517–21522 (2010).

104. E. D. McKenna, S. L. Sarbanes, S. W. Cummings, A. Roll-Mecak, The Tubulin Code, from Molecules to Health and Disease. *Annu. Rev. Cell Dev. Biol.* **39**, 331–361 (2023).

105. F. M. Iseka, *et al.*, Role of the EHD Family of Endocytic Recycling Regulators for TCR Recycling and T Cell Function. *J. Immunol.* **200**, 483–499 (2018).

106. F. E. Rivera-Molina, Z. Xi, E. Reales, B. Wang, D. Toomre, Exocyst complex mediates recycling of internal cilia. *Curr. Biol.* **31**, 5580-5589.e5 (2021).

107. K. K. Rogers, *et al.*, The exocyst localizes to the primary cilium in MDCK cells. *Biochem Bioph Res Co* **319**, 138–143 (2004).

108. S. Fan, *et al.*, Polarity Proteins Control Ciliogenesis via Kinesin Motor Interactions. *Curr. Biol.* **14**, 1451–1461 (2004).

109. M. S. Lutz, R. D. Burk, Primary Cilium Formation Requires von Hippel-Lindau Gene Function in Renal-Derived Cells. *Cancer Res.* **66**, 6903–6907 (2006).

110. F. Mille, *et al.*, The Shh Receptor Boc Promotes Progression of Early Medulloblastoma to Advanced Tumors. *Dev. Cell* **31**, 34–47 (2014).

111. A. S. Pathania, X. Ren, M. Y. Mahdi, G. M. Shackleford, A. Erdreich-Epstein, GRK2 promotes growth of medulloblastoma cells and protects them from chemotherapy-induced apoptosis. *Sci. Rep.* **9**, 13902 (2019).

112. J. K. Ocasio, R. D. P. Bates, C. D. Rapp, T. R. Gershon, GSK-3 modulates SHH-driven proliferation in postnatal cerebellar neurogenesis and medulloblastoma. *Development* **146**, dev177550 (2019).

113. H. Peng, *et al.*, Myomegalin regulates Hedgehog pathway by controlling PDE4D at the centrosome. *Mol. Biol. Cell* **32**, 1807–1817 (2021).

114. X. Ge, *et al.*, Phosphodiesterase 4D acts downstream of Neuropilin to control Hedgehog signal transduction and the growth of medulloblastoma. *eLife* **4**, e07068 (2015).

115. P. A. Northcott, *et al.*, Medulloblastoma Comprises Four Distinct Molecular Variants. *J. Clin. Oncol.* **29**, 1408–1414 (2010).

116. A. Flora, T. J. Klisch, G. Schuster, H. Y. Zoghbi, Deletion of Atoh1 Disrupts Sonic Hedgehog Signaling in the Developing Cerebellum and Prevents Medulloblastoma. *Science* **326**, 1424–1427 (2009).

117. W. Gruber, *et al.*, DYRK1B as therapeutic target in Hedgehog/GLI-dependent cancer cells with Smoothened inhibitor resistance. *Oncotarget* **7**, 7134–7148 (2016).

118. D. W. Ellison, *et al.*, Medulloblastoma: clinicopathological correlates of SHH, WNT, and non-SHH/WNT molecular subgroups. *Acta Neuropathol.* **121**, 381–396 (2011).

119. S. Singh, *et al.*, Zeb1 controls neuron differentiation and germinal zone exit by a mesenchymal-epithelial-like transition. *eLife* **5**, e12717 (2016).

120. J. Aruga, *et al.*, Mouse Zic1 Is Involved in Cerebellar Development. *J. Neurosci.* **18**, 284–293 (1998).

121. W. Wang, T. Wu, M. W. Kirschner, The master cell cycle regulator APC-Cdc20 regulates ciliary length and disassembly of the primary cilium. *eLife* **3**, e03083 (2014).

122. D. Maskey, *et al.*, Cell cycle-dependent ubiquitylation and destruction of NDE1 by CDK5-FBW7 regulates ciliary length. *EMBO J.* **34**, 2424–2440 (2015).

123. D. Zyss, H. Ebrahimi, F. Gergely, Casein kinase I delta controls centrosome positioning during T cell activation. *J. Cell Biol.* **195**, 781–797 (2011).

124. C. Cassioli, C. T. Baldari, A Ciliary View of the Immunological Synapse. *Cells* **8**, 789 (2019).

125. A. Onnis, C. T. Baldari, Orchestration of Immunological Synapse Assembly by Vesicular Trafficking. *Frontiers Cell Dev Biology* **7**, 110 (2019).

126. C. Cassioli, *et al.*, The Bardet-Biedl syndrome complex component BBS1 controls T cell polarity during immune synapse assembly. *J. Cell Sci.* **134** (2021).
