# Supplementary figures and images for: Permanent cilia loss during cerebellar granule cell neurogenesis involves withdrawal of cilia maintenance and centriole capping
Source: Proc Natl Acad Sci U S A. 2024 Dec 20;121(52):e2408083121. doi: 10.1073/pnas.2408083121 (PMC11670249; doi:10.1073/pnas.2408083121)

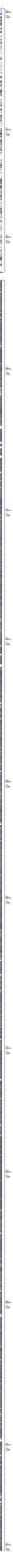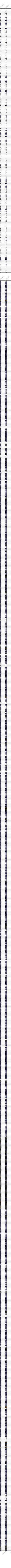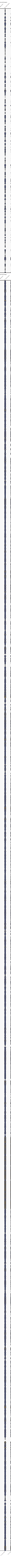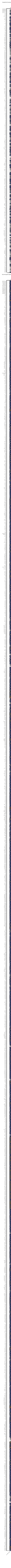

Supplement: Supplementary file 8 — Dataset S07 (PDF) [file pnas.2408083121.sd07.pdf]
